# Accelerating high-throughput virtual screening through molecular pool-based active learning†

**DOI:** 10.1039/d0sc06805e

**Published:** 2021-04-29

**Authors:** David E. Graff, Eugene I. Shakhnovich, Connor W. Coley

**Affiliations:** Department of Chemistry and Chemical Biology, Harvard University Cambridge MA USA; Department of Chemical Engineering, MIT Cambridge MA USA ccoley@mit.edu

## Abstract

Structure-based virtual screening is an important tool in early stage drug discovery that scores the interactions between a target protein and candidate ligands. As virtual libraries continue to grow (in excess of 10^8^ molecules), so too do the resources necessary to conduct exhaustive virtual screening campaigns on these libraries. However, Bayesian optimization techniques, previously employed in other scientific discovery problems, can aid in their exploration: a surrogate structure–property relationship model trained on the predicted affinities of a subset of the library can be applied to the remaining library members, allowing the least promising compounds to be excluded from evaluation. In this study, we explore the application of these techniques to computational docking datasets and assess the impact of surrogate model architecture, acquisition function, and acquisition batch size on optimization performance. We observe significant reductions in computational costs; for example, using a directed-message passing neural network we can identify 94.8% or 89.3% of the top-50 000 ligands in a 100M member library after testing only 2.4% of candidate ligands using an upper confidence bound or greedy acquisition strategy, respectively. Such model-guided searches mitigate the increasing computational costs of screening increasingly large virtual libraries and can accelerate high-throughput virtual screening campaigns with applications beyond docking.

## Abstract 2

Bayesian optimization can accelerate structure-based virtual screening campaigns by minimizing the total number of simulations performed while still identifying the vast majority of computational hits.

## Graphical abstract

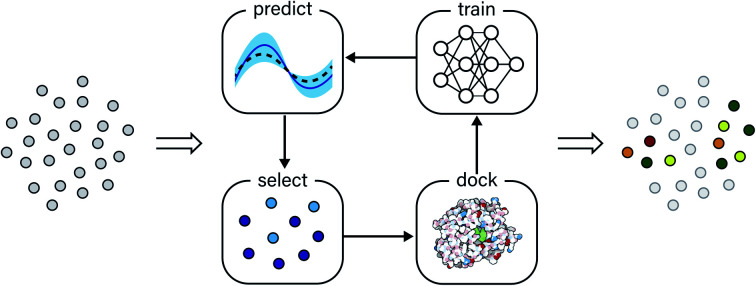

## Introduction

Computer-aided drug design techniques are widely used in early stage discovery to identify small molecule ligands with affinity to a protein of interest.^1,2^ Broadly speaking, these techniques fall into one of two domains: ligand-based or structure-based. Ligand-based techniques often rely on either a quantitative structure–activity relationship (QSAR) or similarity model to screen possible ligands. Both of these ligand-based approaches require one or more previously labeled active/inactive compounds that are typically acquired through physical experimentation. In contrast to ligand-based techniques, structure-based techniques, such as computational docking and molecular dynamics, try to model the protein–ligand interaction and assign a quantitative score intended to correlate with the free energy of binding.^3^ These techniques require a three-dimensional structure of the target protein but do not require target-specific bioactivity data. Scoring functions used in structure-based techniques are typically parameterized functions describing the intra- and intermolecular interactions at play in protein–ligand binding.^3^ As a result, structure-based methods are in principle more able to generalize to unseen protein and ligand structures compared to ligand-based methods. This advantage has been leveraged to discover novel ligand scaffolds in a number of recent virtual screening campaigns.^4^

A typical virtual screening workflow will exhaustively predict the performance of ligands in a virtual chemical library. However, over the past decade, these libraries have grown so large that the computational cost of screening cannot be ignored. For example, ZINC, a popular database of commercially available compounds for virtual screening, grew from 700k to 120M structures between 2005 and 2015 and, at the time of writing, now contains roughly 1 billion molecules.^5,6^ ZINC is not unique in its gargantuan size; other enumerated virtual libraries exist that number well over one billion compounds.^7,8^ Non-enumerated libraries contain an implicit definition of accessible molecules and can be much larger, containing anywhere from 10^10^ to 10^20^ possible compounds.^9–11^ Despite some debate around whether “bigger is better” in virtual screening,^12^ such large virtual libraries are now being used for screening in structure-based drug design workflows.^13–16^ These studies required computational resources that are inaccessible to many academic researchers (*e.g.*, 475 CPU-years in the case of Gorgulla *et al.*^13^). Moreover, this high computational cost makes such a strategy impractical to apply to many distinct protein targets. As virtual libraries grow ever larger, new strategies must be developed to mitigate the computational cost of these exhaustive screening campaigns.

The goal in any virtual screening approach is to find a set of high-performing compounds—herein, computational “hits” with the most negative docking scores—within a significantly larger search space. To restate this formally, we are attempting to solve for the set of top-*k* molecules 
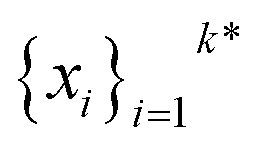
 from a virtual library 
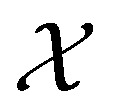
 that maximizes some black-box function of molecular identity 
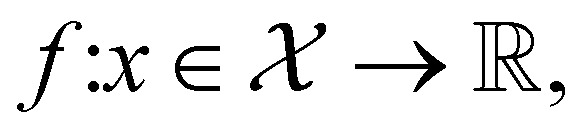
*i.e.*,1
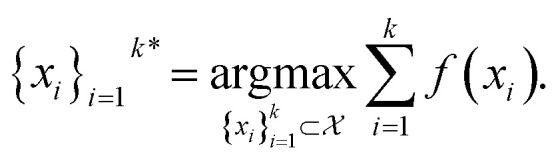


**Fig. 1:**
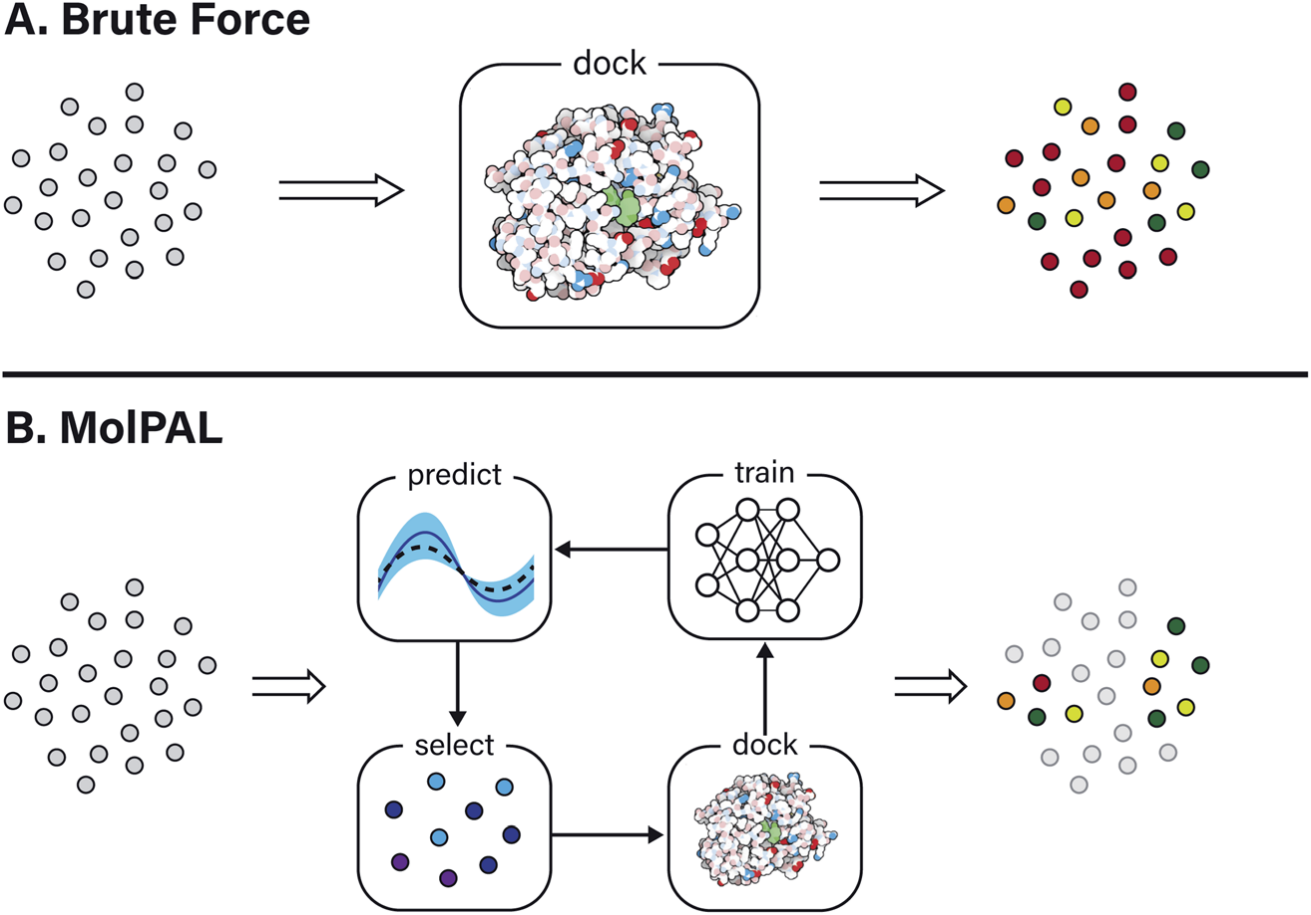
Overview of a computational docking screen using (A) brute force (exhaustive) virtual screening and (B) molecular pool-based active learning (MolPAL), which uses model-guided selection to prioritize the docking of molecules more likely to be scored favorably. Grey circles represent molecules that have not been evaluated.

In this study, the black-box function *f*(*x*) is the negative docking score of a candidate ligand, but other possibilities include the HOMO–LUMO gap of a candidate organic semiconductor, extinction coefficient of a candidate dye, turnover number of a candidate catalyst, *etc.* Brute force searching—screening every molecule in a library indiscriminately—is a straightforward and common strategy employed to solve this type of problem, but it necessarily spends a significant fraction of its time evaluating relatively low-performing compounds (Fig. 1A). However, algorithmic frameworks exist that aim to solve eqn (1) with the fewest number of required evaluations. Bayesian optimization is one such framework that uses a surrogate model trained on previously acquired data to guide the selection of subsequent experiments. We describe Bayesian optimization in more detail in the Methods section below, but we refer a reader to ref. 17 for an in-depth review on the subject.

The application of Bayesian optimization and active learning to physical problems is well-precedented, *e.g.*, with applications to materials design,^18–21^ bioactivity screening,^22–24^ and molecular simulations.^25–27^ These examples showcase the significant efficiency gains made possible through use of model-guided experimentation. However, examples of active learning for structure-based virtual screening are more limited. Compared to the previous examples, structure-based drug design involves search spaces of millions to billions of organic small molecules with simulations that are significantly cheaper than the electronic structure or molecular dynamics simulations from the above examples. The large, discrete, and not explicitly combinatorial nature of the design space coupled with the comparably cheap objective function makes this a relatively unexplored setting for Bayesian optimization.

Active learning applied to computational docking, “iterative docking,” has previously been explored in the context of a binary classification formulation during the QSAR modelling step using fingerprint-based surrogate models.^28–30^ While the specifics of each approach varied, these studies shared similar acquisition strategies *via* the docking of new molecules classified as “hits” by their trained QSAR model. Our work seeks to expand upon these initial studies, particularly by examining the effects of surrogate model architecture, acquisition strategy, and batch size have on optimization performance. Moreover, our work treats docking score as a continuous variable, so our surrogate model follows a regression formulation, as opposed to the binary classification formulation of the previous studies. Lyu *et al.* observed correlations between the success rates of experimental validation and computed docking scores,^14^ suggesting that preserving this information during model training may improve the surrogate model's ability to prioritize molecules that are more likely to be validated as active. Recent work by Pyzer-Knapp^31^ also looked at such an approach using a Gaussian process (GP) surrogate model along with a parallel and distributed Thompson sampling acquisition strategy;^32^ this strategy is well-suited to small design spaces (*e.g.*, thousands of compounds) but does not scale well to millions of acquired data points due to the cost of GP training.^33^ Contemporary studies published in the months following the release of this manuscript's preprint have also looked at the use of active learning to accelerate docking-based virtual screening.^34–36^

In this work, we leverage Bayesian optimization algorithms for docking-based virtual screening in a manner that decreases the computational cost of using docking to identify the majority of top-scoring compounds in virtual libraries by over an order of magnitude (Fig. 1B). We demonstrate that surrogate machine learning models can prioritize the screening of molecules that are associated with better docking scores, which are presumed to be more likely to validate experimentally.^14^ We analyze a variety of different model architectures and acquisition functions that one can use to accelerate high-throughput virtual screening using Bayesian optimization. Specifically, we test random forest (RF), feedforward neural network (NN), and directed-message passing neural network (MPN) surrogate models along with greedy, upper confidence bound (UCB), Thompson sampling (TS), expectation of improvement (EI), and probability of improvement (PI) acquisition strategies in addition to various acquisition batch sizes. We study these optimization parameters on multiple datasets of protein–ligand docking scores for compound libraries containing roughly ten thousand, fifty thousand, two million, and one hundred million molecules. We perform these studies using MolPAL, an open source software which we have developed and made freely available.

## Results

### Small virtual libraries

**Fig. 2:**
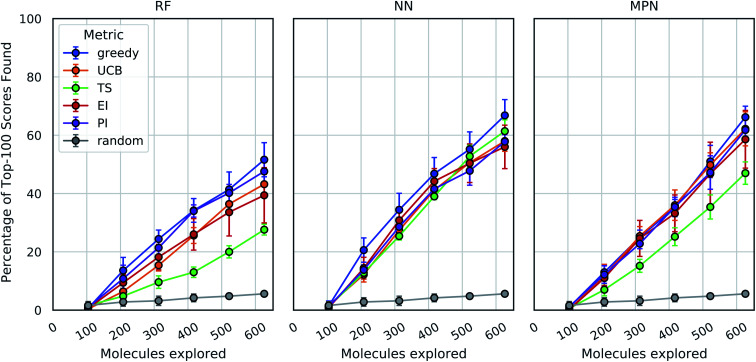
Bayesian optimization performance on Enamine 10k screening data as measured by the percentage of top-100 scores found as function of the number of ligands evaluated. Each trace represents the performance of the given acquisition metric with the surrogate model architecture corresponding the chart label. Each experiment began with random 1% acquisition (*ca.* 100 samples) and acquired 1% more each iteration for five iterations. Error bars reflect ± one standard deviation across five runs. RF, random forest; NN, feedforward neural network; MPN, message passing neural network; UCB, upper confidence bound; TS, Thompson sampling; EI, expected improvement; PI, probability of improvement.

As an initial evaluation of the experimental efficiency Bayesian optimization can provide, we generated a dataset containing docking scores from 10 560 compounds (Enamine's smaller Discovery Diversity Collection, “Enamine 10k”) docked against thymidylate kinase (PDB ID: 4UNN^37^) using AutoDock Vina.^38^ Data acquisition was simulated as the iterative selection of 1% of the library (*ca.* 100 molecules) repeated five times after initialization with a random 1% subset for a total acquisition of 6% of the library. We first looked at a random forest (RF) operating on molecular fingerprints as our surrogate model along with a greedy acquisition strategy. This combination yields clear improvement over the random baseline, representative of a brute-force search, when looking at the percentage of top-100 (*ca.* top-1%) scores in the full dataset found by MolPAL (Fig. 2, left panel). The greedy strategy finds, on average, 51.6% (±5.9) of the top-100 scores in the full dataset when exploring only 6% of the pool.

We define the enrichment factor (EF) as the ratio of the percentage of top-*k* scores found by the model-guided search to the percentage of top-*k* scores found by a random search for the same number of objective function calculations. The random baseline finds only 5.6% of the top-100 scores in the Enamine 10k dataset, thus constituting an EF of 9.2 for the greedy random forest combination. A UCB acquisition metric, yields similar, albeit slightly lower, performance with an EF of 7.7. Surprisingly, the other optimistic acquisition metric we tested, Thompson sampling (TS), does show an improvement over the random baseline but is considerably lower than all other metrics (EF = 4.9). We attribute this lower performance to the large uncertainties in the RF model, which cause the distributions of predicted scores to overlap between molecules and make the Thompson sampling strategy behave similarly to random sampling.

We next assessed the effect of architecture for the surrogate model. Using a fully connected feedforward neural network (NN) operating on molecular fingerprints, we observed an increase in performance for all acquisition metrics (Fig. 2, middle panel). With the NN model, the least performant acquisition strategy (56.0% with EI) was more powerful than the best performing strategy with the RF model (51.6% with greedy acquisition). The greedy acquisition metric remains the best performing for the NN model, achieving 66.8% of top-100 scores found for an EF of 11.9. The third and final architecture examined is a message passing neural network model (MPN), using the D-MPNN implementation by Yang *et al.*^39^ The MPN model resulted in comparable performance to the NN model (Fig. 2, right panel), with slight improvement for some metrics. However, the highest performance observed, 66.2% (EF = 11.8) with greedy acquisition, is statistically identical to the best NN result.

**Fig. 3:**
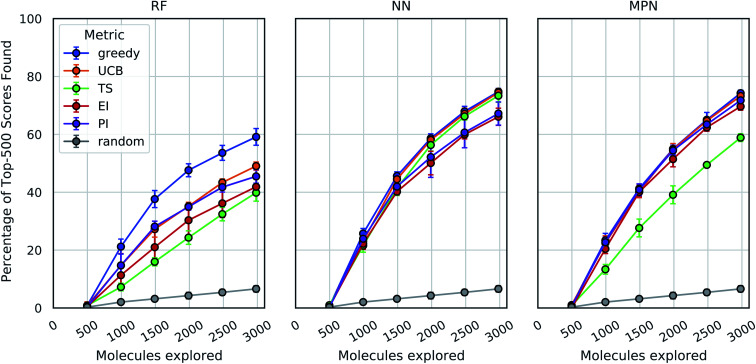
Bayesian optimization performance on Enamine 50k screening data as measured by the percentage of top-500 scores found as function of the number of ligands evaluated. Each trace represents the performance of the given acquisition metric with the surrogate model architecture corresponding the chart label. Each experiment began with random 1% acquisition (*ca.* 500 samples) and acquired 1% more each iteration for five iterations. Error bars reflect ± one standard deviation across five runs.

These analyses were repeated for Enamine's larger Discovery Diversity Collection of 50 240 molecules (“Enamine 50k”) also against 4UNN with the same docking parameters (Fig. 3). We again took 1% of the library as our initialization with five subsequent exploration batches of 1% each. All of the trends remained largely the same across acquisition metrics and model architectures; we observed comparable quantitative performance for every surrogate model/acquisition metric combination as compared to the smaller library. For example, the RF model with a greedy acquisition strategy now finds 59.1% (±2.9) of the top-500 scores (*ca.* top-1%) using the Enamine 50k library *vs.* the 51.6% of the top-100 scores (*ca.* top-1%) when using the Enamine 10k library. There was little difference between the results of the NN and MPN models on the Enamine 50k results, which find 74.8% and 74.2% of the top-500 scores after exploring just 6% of the library, respectively. These values represent enrichment factors of 12.5 and 12.4, respectively, over the random baseline.

### Enamine HTS collection

Encouraged by the significant enrichment observed with the Enamine 10k and 50k datasets, we next tested Enamine's 2.1 million member HTS Collection (“Enamine HTS”) – a size more typical of a high-throughput virtual screen. We again use 4UNN and Autodock Vina to define the objective function values. With this larger design space, acquisitions of 1% represent a significant number of molecules (*ca.* 21 000); therefore, we also sought to reduce exploration size. Given the strong performance of the greedy acquisition metric and its simplicity (*i.e.*, lack of a requirement of predicted variances), we focus our analysis on this metric alone.

**Fig. 4:**
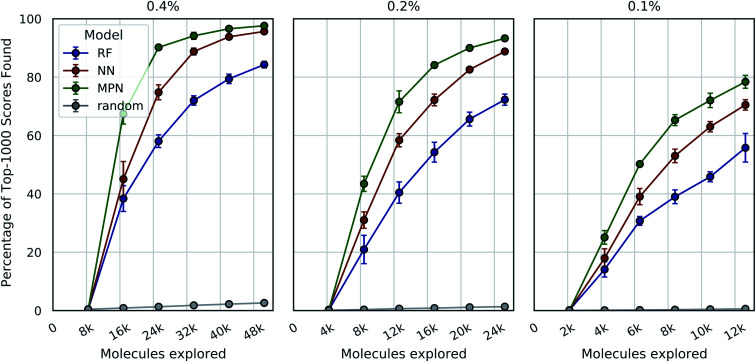
Bayesian optimization performance on Enamine HTS docking data as measured by the percentage of top-1000 scores found as function of the number of ligands evaluated. Each trace represents the performance of the given model with a greedy acquisition strategy. Chart labels represent the fraction of the library acquired in the random initial batch and the five subsequent exploration batches. Error bars reflect ± one standard deviation across five runs.

We tested three different batch sizes for initialization and exploration, with five exploration batches, as in our above experiments. Using a batch size of 0.4% for a total of 2.4% of the pool, we observed strong improvement over random exploration for all three models using the greedy metric in terms of the fraction of the top-1000 (top-0.05%) scores found (Fig. 4, left panel). With a 0.4% batch size, the random baseline finds only 2.6% of the top-1000 scores, whereas the RF, NN, and MPN models find 84.3% (EF = 32.4), 95.7% (EF = 36.8), and 97.6% (EF = 40.7) of the top-1000 scores, respectively. Lowering the total exploration size by half so that 0.2% of the library is acquired at each iteration (a total of 1.2% of the library) reduces the overall performance of each model, but the drop in performance is not commensurate with the decrease in performance of the random baseline (Fig. 4, middle panel). The MPN model is shown to be the most robust to the decrease in batch size, identifying 93.3% of the top-1000 scores after exploring just 1.2% of the design space for an enrichment factor of 77.8. Similarly, reducing the batch size further to 0.1% affects the random baseline to a greater extent than any active learning strategy (Fig. 4, right panel). Here, the random baseline finds only 0.6% of the top-1000 scores but the MPN model with greedy acquisition finds 78.4% of the top-1000 scores, representing an enrichment factor of 131. This growth in enrichment factor as exploration fraction decreases holds for other, non-greedy acquisition metrics (Tables S3–S5†).

### Ultra-large libraries

One goal of the Bayesian optimization framework in our software, MolPAL, is to scale to even larger chemical spaces. A two million member library is indeed a large collection of molecules, but screens of this size are compatible with standard high-performance computing clusters. Our final evaluation sought to demonstrate that MolPAL can make virtual screens of ≥10^8^-member libraries accessible to researchers using modest, shared clusters. We turned to a recent study by Lyu *et al.* that screened over 99 million and over 138 million readily accessible molecules against AmpC β-lactamase (PDB ID: 1L2S) and the D_4_ dopamine receptor (PDB ID: 5WIU), respectively, using DOCK3.7.^14^ The full datasets containing all of the molecules that were successfully docked against 1L2S and 5WIU (“AmpC” and “D_4_”, respectively) are used as the ground truth for the appropriate experiments.^40,41^ We first measure our algorithm's performance as a function of the top-50 000 (top-0.05%) scores found in the AmpC dataset for all three models using acquisition sizes of 0.4%, 0.2%, or 0.1%.

**Fig. 5:**
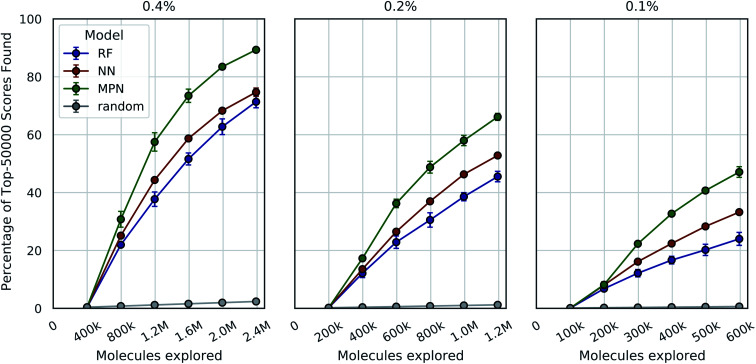
Bayesian optimization performance on AmpC docking data (99.5M) as measured by the percentage of top-50 000 scores found as function of the number of ligands evaluated. Each trace represents the performance of the given model with a greedy acquisition strategy. Chart labels represent the fraction of the library taken in both the initialization batch and the five exploration batches. Error bars reflect ± one standard deviation across three runs.

We see a similar qualitative trend for the AmpC dataset as for all previous experiments: namely, that the use of Bayesian optimization enables us to identify many of the top-performing compounds after exploring a small fraction of the virtual library, even when using a greedy acquisition metric (Fig. 5). For the 0.4% batch size experiments, the MPN model finds 89.3% of the top-50 000 (*ca.* top-0.05%) scores after exploring 2.4% of the total pool (EF = 37.2). Decreasing the batch size to 0.2% led to a recovery of 66.2% (EF = 55.2), and further decreasing the batch size to 0.1% resulted in 47.1 recovery (EF = 78.5). The NN and RF surrogate models were not as effective as the MPN, but still led to significant enrichment above the baseline. Results for additional acquisition functions can be found in Tables S6–S8.† The UCB acquisition metric led to notable increases in the performance of the MPN model for all batch sizes, finding 94.8% (EF = 39.5), 77.5% (EF = 64.6), and 48.7% (EF = 81.2) of the top-50 000 scores for the 0.4%, 0.2%, and 0.1% batch sizes, respectively. The UCB metric similarly outperformed the greedy metric on the D_4_ dataset with the MPN model, finding 84.3% (EF = 35.1), 68.6% (EF = 57.2), and 52.8% (EF = 88.0) of the top-50 000 scores for batch sizes 0.4%, 0.2%, and 0.1% batch sizes, respectively (Fig. S2†). However, UCB was not consistently superior to greedy acquisition for other model architectures.

It is worth commenting on the differences in quantitative enrichment factors reported for the AmpC data and Enamine HTS data. There are at least two differences that preclude a direct comparison: (1) the top-*k* docking scores in the AmpC data were generated by DOCK and span a range of −118.83 to −73.99. This is compared to docking scores from the Enamine HTS collection dataset calculated with AutoDock Vina, where the top-*k* docking scores range from −12.7 to −11.0. The lower precision of AutoDock Vina scores makes the top-*k* score metric more forgiving (discussed later in the Limitations of evaluation metrics subsection). (2) The chemical spaces canvassed by both libraries are different. This will necessarily impact model performance and, by extension, optimization performance.

### Single-iteration active learning

**Fig. 6:**
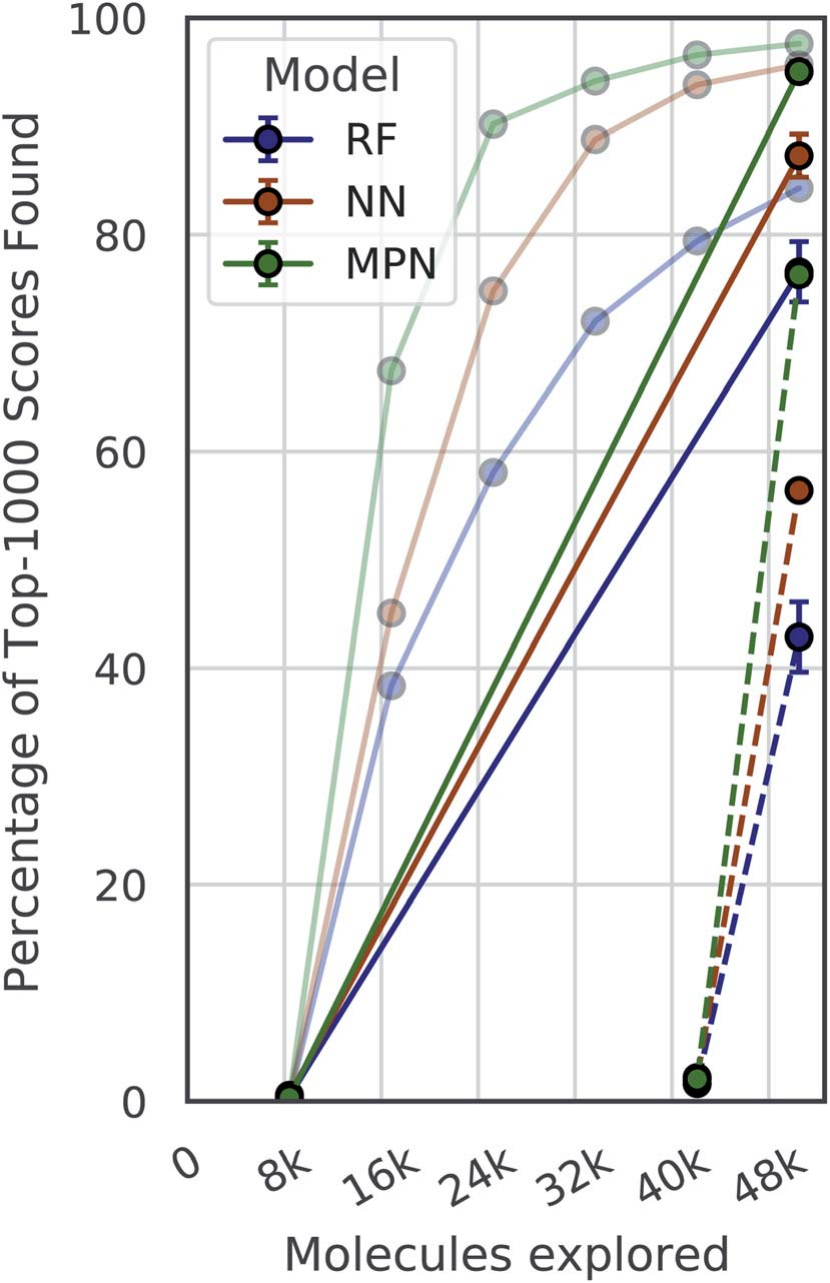
Single-iteration results on Enamine HTS docking data (2.1M) with greedy acquisition. Solid traces: initialization batch size of 0.4% of pool and exploration batch size of 2% of pool. Dashed traces: initialization batch size of 2% of pool and exploration batch size of 0.4% of pool. Error bars reflect ± one standard deviation across three runs. Faded traces: reproduced active learning data from 0.4% experiments (error bars omitted).

A critical question with these experiments is the importance of the active learning strategy. From the Enamine HTS data (Fig. 4), we observe a sharp increase in the percentage of top-1000 scores found after the first exploration batch (*e.g.*, from 0.4% to 67.4% for the MPN 0.4% acquisition), suggesting that the randomly selected initial batch is quite informative. We look at “single-iteration” experiments, where the first batch is selected randomly and the second (and final) batch is selected according to our acquisition function (Fig. 6). Selecting all 42 000 molecules at once after training on the initial 8400 molecules results in finding 92.7% (±1.5) of the top-1000 scores with an MPN model after exploring 2.4% of the library (EF = 38.6). This is slightly (but significantly) lower than the active learning case with an MPN model, which finds 97.6% of the top-1000 scores (EF = 40.7). Using an NN or an RF model, the differences in active learning *versus* the single-iteration case are more pronounced. We also test the setting where the initial batch consists of 42 000 molecules and the single active learning iteration only selects 8400. The smaller acquisition size for the second batch results in considerably lower MPN performance, finding only 76.2% of the top-1000 scores (EF = 31.8). This is worse than any model we tested with an active learning-based strategy at the same number of function evaluations. The less flexible NN and RF models suffer even more pronounced drops in performance with a large initial batch and small exploration batch.

### Dynamic convergence criterion

**Fig. 7:**
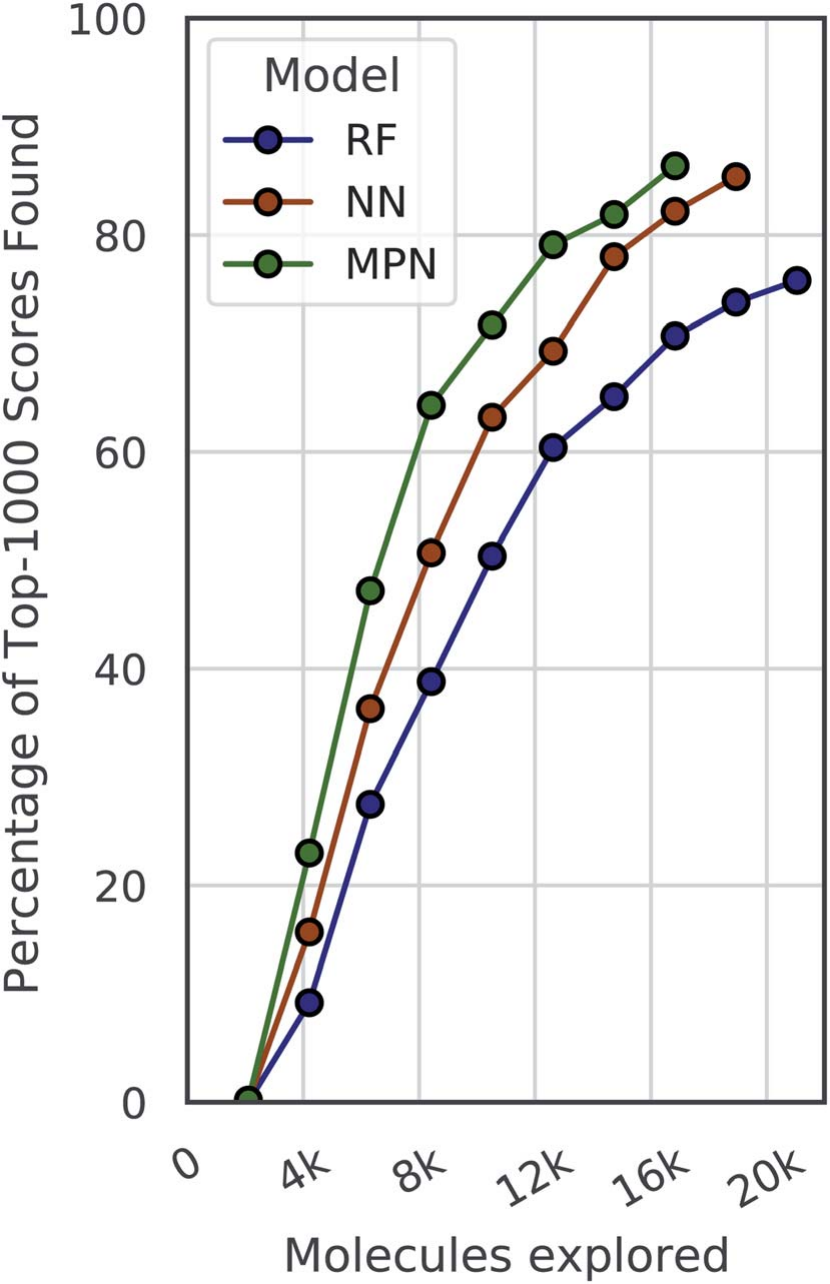
Optimization performance on Enamine HTS docking data (2.1M) using a greedy acquisition function when convergence is defined based on the degree of improvement of top-1000 scores between two consecutive iterations not exceeding 0.01. A 0.1% batch size was used for both initialization and exploration.

Our evaluations so far have demonstrated reliable performance of MolPAL using a fixed exploration strategy (*i.e.*, number of iterations). However, we will typically not know *a priori* what an appropriate total exploration size is. We therefore define a convergence criterion for the Enamine HTS dataset that is satisfied when the fractional difference between the current average of the top-1000 scores and the rolling average of the top-1000 scores from the previous three iterations, corresponding to the top 0.05% of compounds, is less than a fixed threshold, here 0.01. Fig. 7 illustrates the use of this convergence criterion using a 0.1% batch size (*ca.* 2100 molecules) with a greedy acquisition metric. We find that not only do the higher capacity models converge sooner (MPN > NN > RF) but they also converge to a higher percentage of top-1000 scores found (86.4%, 85.4%, and 75.8%, respectively).

### Chemical space visualization

**Fig. 8:**
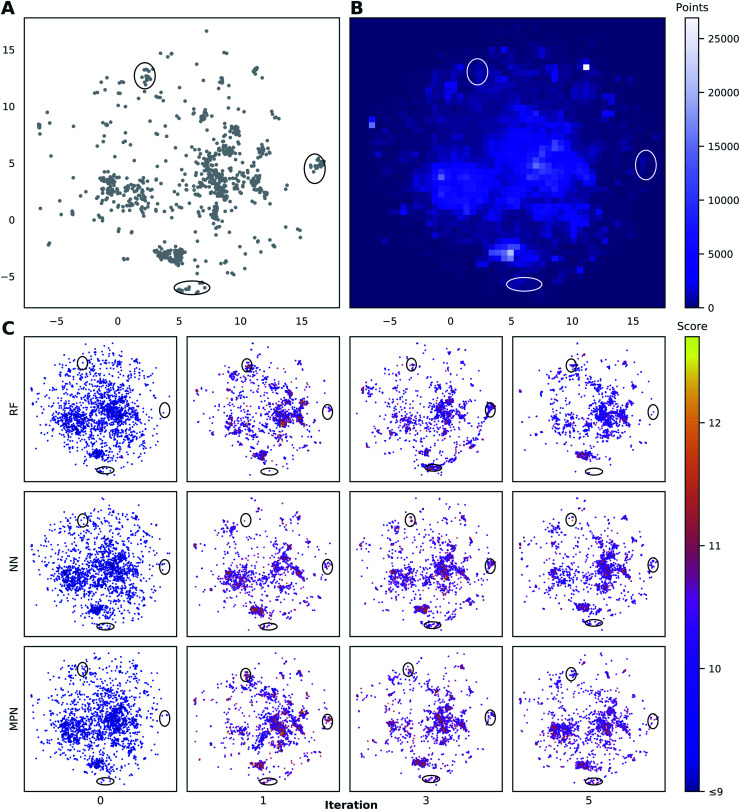
Visualization of the chemical space in the Enamine HTS library (2.1M molecules) using UMAP embeddings of 2048-bit atom-pair fingerprints trained on a random 10% subset of the full library. (A) Embedded fingerprints of the top-1000 scoring molecules. (B) 2-D density plot of the embedded fingerprints of the full library. (C) Embedded fingerprints of the molecules acquired in the given iteration using a greedy acquisition metric, 0.1% batch size, and specified surrogate model architecture z-ordered by docking score. Circled regions indicate clusters of high-scoring compounds in sparse regions of chemical space. Color scale corresponds to the negative docking score (higher is better). *x*- and *y*-axes are the first and second components of the 2D UMAP embedding and range from −7.5 to 17.5.

To visualize the set of molecules acquired during the Bayesian optimization routine, we projected the 2048-bit atom-pair fingerprints of the Enamine HTS library into two dimensions using UMAP^42^ (Fig. 8 and S23†). The embedding of the library was trained on a random 10% subset of the full library and then applied to the full library. Comparing the location of the top-1000 molecules (Fig. 8A) to the density of molecules in the entire 2M library (Fig. 8B) reveals several regions of high-performing compounds located in sparse regions of library chemical space, of which we focus on three (black ellipses). We plot the embedded fingerprints of the molecules acquired in the zeroth (random), first, third, and fifth iterations of a 0.1% batch size greedy search with the three surrogate models (Fig. 8C) to observe how each model guides exploration of the same chemical space. This analysis is qualitative in nature because UMAP projections are highly dependent on the set of hyperparameters used to train the embedding. However, these plots are helpful in illustrating how the three surrogate models differ in which regions they choose to sample from. In addition, this visualization allows us to observe how each search evolves throughout the optimization. For example, the MPN model begins to sample more heavily from the dense region of chemical space around (0, 2.5) (Fig. 8B) as the optimization proceeds.

## Discussion

### Application to other optimization objectives

All of the above datasets involve docking scores obtained either through AutoDock Vina or DOCK3.7. We performed similar retrospective studies using a dataset from Yang *et al.*^34^ that contains the same 100M molecules docked against AmpC using Glide SP rather than DOCK3.7 (“AmpC Glide”). For this new dataset, we still observe significant performance gains over the random baseline, identifying 77.1% of the top-50 000 scores after sampling only 2.4% of the total library (EF = 32.1) using an MPN surrogate model and UCB acquisition metric (Fig. S3†). We also examined the application of MolPAL to the Harvard Clean Energy Project dataset (“HCEP”) of 2.4M organic photovoltaic materials and their predicted power conversion efficiencies (PCE)^43^ as an example of a non-docking objective function. With an MPN model and a UCB acquisition metric, MolPAL identifies 91.1% of the top-1000 PCEs after acquiring only 4.4% of the overall library (EF = 20.7) (Fig. S5†).

### Effect of acquisition strategy on performance

An interesting result from these experiments was the consistently strong performance of the greedy acquisition metric. This is surprising given the fact that the greedy metric is, in principle, purely exploitative and vulnerable to limiting its search to a single area of the given library's chemical space. Prior work in batched Bayesian optimization has focused on developing strategies to prevent batches from being homogeneous, including use of an inner optimization loop to construct batches one candidate at a time.^44,45^ Despite this potential limitation of the greedy acquisition metric, it still leads to adequate exploration of these libraries and superior performance to metrics that combine some notion of exploration along with exploitation (*i.e.*, TS, EI, PI). One confounding factor in this analysis is that methods used for uncertainty quantification in regression models are often unreliable,^46^ which may explain the poorer empirical results when acquisition depends on their predictions. While the experiments using the AmpC, AmpC Glide, D_4_, and HCEP datasets demonstrated optimal performance with an MPN model and UCB metric, the greedy metric still performed well in each of these experiments and outperformed the TS metric.

To investigate whether the UCB metric's performance on the ultra-large docking datasets was a function of the size of the dataset, we performed subsampling experiments. Five random subsets of 2M SMILES strings and their associated docking scores were taken from the full AmpC dataset. MolPAL was applied to each library using an MPN model with a greedy, UCB, or TS metric and 0.4%, 0.2%, or 0.1% acquisition batch size. The trend among greedy, UCB, and TS acquisition metrics remains largely the same (Fig. S4†) as that of the full dataset (Fig. 5), suggesting that library size may not be as important as the objective function itself in determining the optimal acquisition function.

### Effect of library size

The principal takeaway from our results on different library sizes is that Bayesian optimization is not simply a viable technique but an effective one in all of these cases. Though it is difficult to quantitatively compare algorithm performance on each dataset due to their differing chemical spaces, the impact of library size on the optimization is still worth commenting on. We observe the general trend in our data that, as library size increases, so too does top-*k* performance given a constant fractional value for *k*, even when decreasing relative exploration size. We anticipate that this is due in part to the greater amount of training data that the surrogate model is exposed to over the course of the optimization. Despite the relative batch size decreasing, the absolute number of molecules taken in each batch and thus the number data points to train on increases from roughly 500 to nearly 8400 when moving from the Enamine 50k dataset (1% batch size) to the Enamine HTS dataset (0.4% batch size). We analyzed the mean-squared error (MSE) of MPN model predictions on the entire Enamine 10k, Enamine 50k, and HTS datasets after initialization with random 1% batches; the MSE decreased with increasing library size (Table 1). This trend suggests that the overall “diversity” of the chemical library is not increasing at the same rate as the size, *i.e.*, with a fixed fractional training set size, it is easier to achieve high correlation on larger libraries. As a result, the surrogate model is better able to prioritize the acquisition of high-performing molecules.

**Table 1:** Predictive performance with an MPN model trained on a random 1% batch of the corresponding library

Library	Total size	Training size	MSE (↓)	Spearman's *ρ* (↑)
Enamine 10k	10 560	106	0.506	0.454
Enamine 50k	50 240	503	0.258	0.789
Enamine HTS	2 141 514	21 416	0.146	0.919

### Consistency across repeated trials

**Fig. 9:**
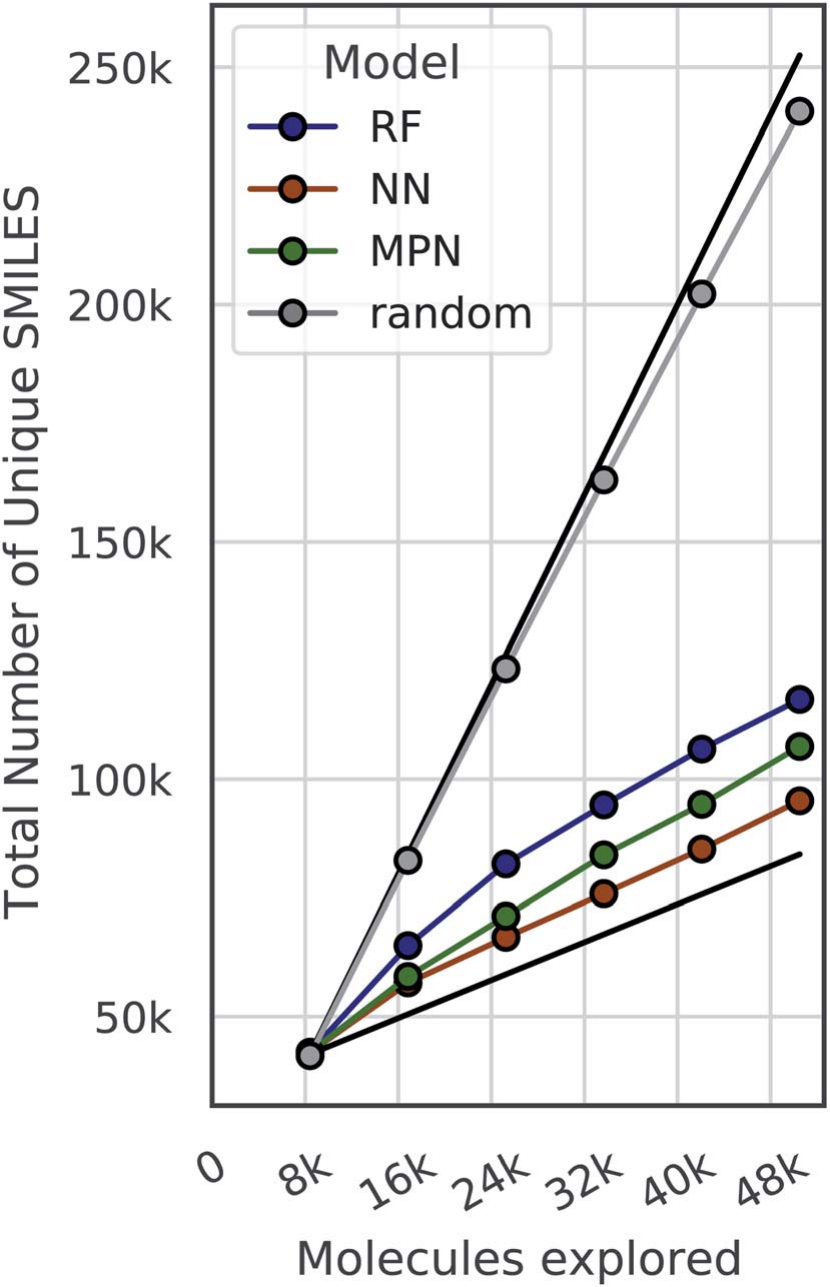
The total number of unique SMILES strings acquired across 5 repeated greedy optimizations with a 0.4% batch size on the Enamine HTS docking dataset (2.1M). The top black line is the theoretical maximum (*i.e.*, repeated trials select distinct subsets of molecules to evaluate), and the bottom black line is the theoretical minimum (*i.e.*, repeated trials select identical subsets of molecules to evaluate).

Bayesian optimization can be prone to large deviations across repeated trials, but our results showed consistent performance across all datasets and configurations (Tables S1–S8†). To investigate whether the consistency in performance is a result of consistency in the exact molecular species selected, we calculate the total number of unique SMILES strings acquired across all repeated experiments as a function of optimization iteration (Fig. 9, S13, and S14†). Comparing these results to both the theoretical maximum (each trial acquiring a completely unique subset of molecules at each iteration) and the theoretical minimum (each trial acquiring an identical subset of molecules at each iteration after the initialization) approximates the degree to which each repeat explores the same or different regions of chemical space. Traces closer to the maximum would indicate that each experiment is exploring a unique subset of highly performing molecules, and traces closer to the minimum signal the opposite: that each experiment is converging towards the same regions of chemical space. Our data are most consistent with the latter, suggesting that each trial is converging towards the same optimal subset of the library regardless of its initialization. We hypothesize that this is due to the relatively smooth structure–property landscapes present in these datasets and lack of statistical uncertainty. Characterizing the roughness of the structure-score landscape and whether active compounds that are found in clusters of inactive compounds are harder to find is an area of ongoing research.

### Limitations of evaluation metrics

In this study, three separate evaluation criteria were used to assess performance: the average top-*k* docking score identified, the fraction of top-*k* SMILES identified, and the fraction of top-*k* scores identified throughout the optimization campaign (calculation details are provided in the Methods section below). The average metric is sensitive to the scale and distribution of scores, making direct comparison between datasets challenging. The top-*k* SMILES metric asks whether a specific set of molecules is selected by the algorithm, which can be overly strict if multiple molecules have identical performance (*i.e.*, the same score) but are not within the top-*k* due to arbitrary data sorting (Fig. S6–S8†). The top-*k* score metric overcomes this limitation by assigning equal weight to each molecule with the same score regardless of its specific position in the sorted dataset. As a result, this makes the scores metric more forgiving for datasets with smaller ranges and lower precision (*e.g.*, those calculated using AutoDock Vina with a precision of 0.1) than those with larger ranges and higher precision (*e.g.*, those calculated using DOCK with a precision of 0.01). In contrast to the average top-*k* score, however, this metric does not reward “near-misses”, for example, identifying the *k* + 1 ranked molecule with a nearly identical score to the *k*-th molecule.

### Optimal batch size for active learning

The number of molecules selected at each iteration represents an additional hyperparameter for Bayesian optimization. In one limit, Bayesian optimization can be conducted in a purely sequential fashion, acquiring the performance of a single molecule each iteration. Fully sequential learning would offer the most up-to-date surrogate model for the acquisition of each new point but it would also be extremely costly to continually retrain the model and perform inference on the entire candidate pool. While some studies look at the prospect of “warm starting” to accelerate neural network training,^47^ and while we also examined using online retraining only on the newly acquired batch (Fig. S15–S22†), the cost of repeated inference cannot be lowered. In the other limit, molecules would be selected in a single iteration, which can lead to suboptimal performance depending on the acquisition size (Fig. 6). Finding a principled balance between these two without resorting to empirical hyperparameter optimization is an ongoing challenge.^48,49^ In each of our experiments, the relative batch size was held constant at one sixth of the total exploration size. Future performance engineering work will seek to examine the effects of dynamic batch sizes in batched optimization. Note that overall batch diversity is another consideration in batched Bayesian optimization. While selected batches in this study did not appear to suffer from homogeneity, previous approaches to improve batch diversity could be explored as well.^32,45,50^

### Cost of surrogate model (re)training

The question of optimal batch size cannot be decoupled from the computational cost of model retraining and inference. Throughout these studies, we have focused only on the number of objective function calculations necessary to achieve a given level of performance. While objective function calculation is significantly more expensive than the cost of model training and inference, inference costs scale linearly with the size of the dataset and contribute to the overall cost of our algorithm.

The MPN model was shown to be superior in the largest datasets, but its costs are markedly higher than those of the fingerprint-based NN model. The tradeoff between sample efficiency (number of objective function calculations) and surrogate model costs (training and inference) should be balanced when selecting a model architecture. In our evaluation, the costs of the MPN and NN cannot be directly compared due to differences in their implementation and extent of both parallelization and precalculation. For more details, see the software design subsection in the ESI.† An additional choice when seeking to limit surrogate model costs is whether to train the model online with newly acquired data or fully retrain the model at each iteration with all acquired data. We examined this possibility, but online learning lead to consistently lower performance in our experiments (Tables S1–S8†).

## Conclusion

In this work, we have demonstrated the application of Bayesian optimization to the prioritization of compounds for structure-based virtual screening using chemical libraries ranging in size from 10k to 138M ligands. A thorough evaluation of different acquisition metrics and surrogate model architectures illustrates (1) that active learning is able to identify the vast majority of top-scoring compounds with significantly reduced cost, (2) that a greedy acquisition metric still results in strong performance, though UCB is better in certain cases, and (3) that the message passing neural network model outperforms fingerprint-based feedforward neural network or random forest models. In the AmpC dataset, where a 100M member library was docked against 1L2S by Lyu *et al.*, we identify 89.3% of the top-50 000 scoring ligands with a 40-fold reduction in the number of docking calculations using a greedy acquisition metric and 94.8% using a UCB acquisition metric.

We believe that this model-guided approach to compound prioritization should become standard practice as a drop-in replacement for exhaustive high-throughput virtual screening when the exact set of top-*k* compounds is not needed. Moreover, this approach is also relevant to *experimental* high-throughput screening, an expensive and important tool for challenging drug discovery problems.^51^ Future work will seek to extend the open source MolPAL software package and leverage it in a prospective manner to greatly accelerate a structure-based virtual screen of the Enamine REAL database. We also hope to expand MolPAL beyond the initial software detailed in this report with the addition of new surrogate model architectures, the inclusion of improved uncertainty estimation techniques, and the expansion to other forms of virtual discovery, *i.e.*, other objective functions. Finally, we envision that, in addition to accelerating the practice of virtual screening, MolPAL will increase its accessibility through the pipelining of the entire virtual screening process behind a common interface.

## Methods

### Batched Bayesian optimization

Bayesian optimization is an active learning strategy that iteratively selects experiments to perform according to a surrogate model's predictions, often using machine learning (ML) models. In the context of this work, the Bayesian optimization was performed on a discrete set of candidate molecules, herein referred to as a “pool” or virtual library, and points were acquired in batches rather than one point at a time. Batched Bayesian optimization begins by first calculating the objective function *f*(*x*) for a set of *n* random points {*x*}_*i*=1_^*n*^ within a pool of points 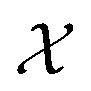. The objective function values for these points are calculated, in this study as docking scores from AutoDock Vina, and the corresponding tuples {(*x*_*i*_, *f*(*x*_*i*_))}_*i*=1_^*n*^ are stored in the dataset 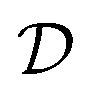. A surrogate model *f̂*(*x*) is then trained on these data and, along with the current maximum objective function value f*, is passed to an acquisition function *α*(*x*; *f̂*, f*), which calculates the utility of evaluating the objective function at the point *x*. Utility may be measured in a number of ways: the predicted objective function value, the amount of information this new point will provide the surrogate model, the likelihood this point will improve upon the current maximum, *etc.* See ref. 52 for a detailed discussion of various acquisition functions. The set of *b* points with the largest sum of utilities 
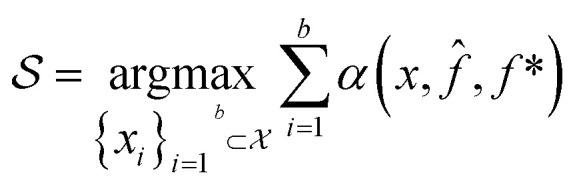
 is then selected, or “acquired”. The set of objective function values corresponding to these points is calculated 
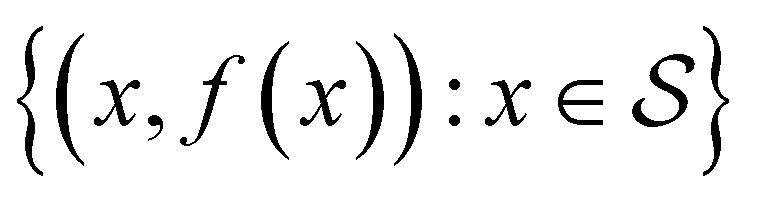
 and used to update the dataset 
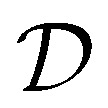
. This process is repeated iteratively until a stopping criterion is met (*e.g.*, a fixed number of iterations or a lack of sufficient improvement).
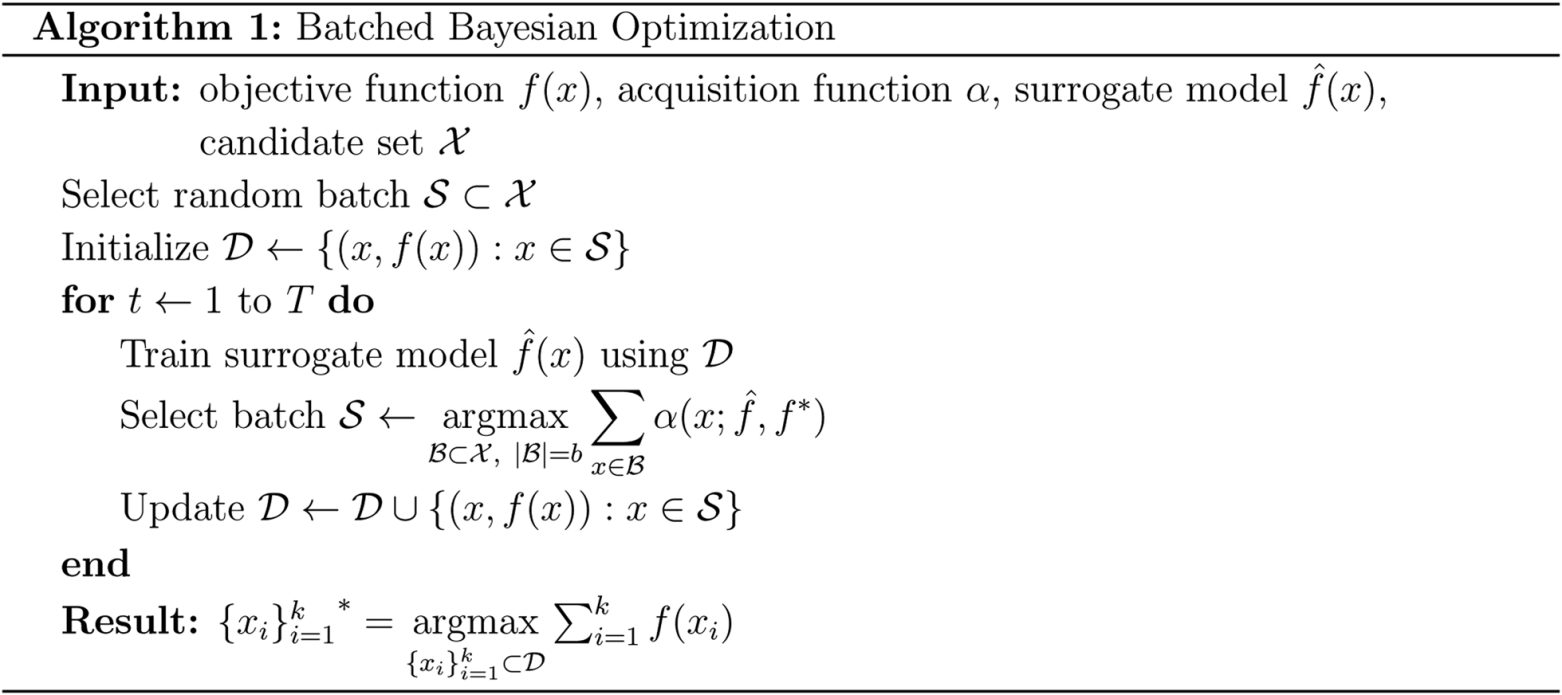


### Acquisition metrics

The following acquisition functions were tested in this study:
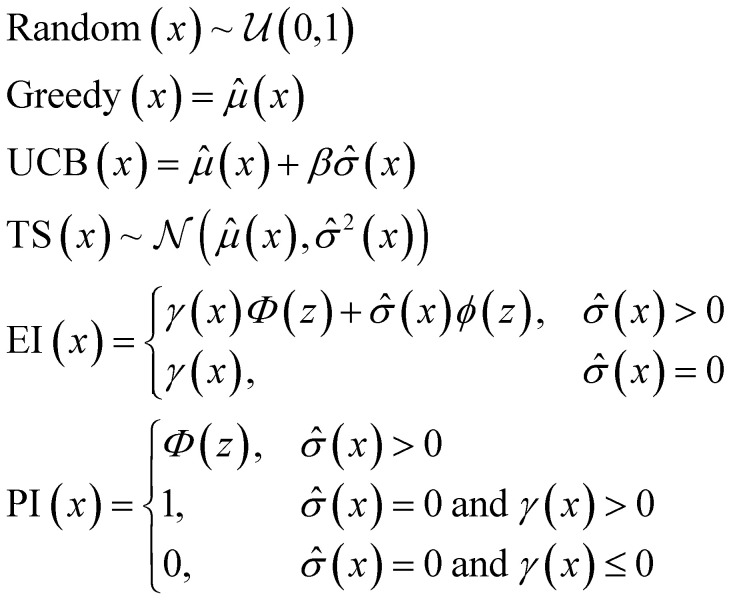
where: *γ*(*x*): = *μ̂*(*x*) − f* + *ξ*; 
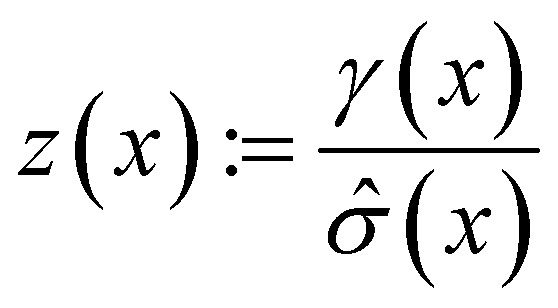
; *μ̂*(*x*) and *σ̂*^2^(*x*) are the surrogate model predicted mean and uncertainty at point *x*, respectively; *Φ* and *ϕ* are the cumulative distribution function and probability density function of the standard normal distribution, respectively; and f* is the current maximum objective function value. For the experiments reported in the paper, we used *β* = 2 and *ξ* = 0.01.

### Surrogate models

In the context of our study, the surrogate modelling step involved training a machine learning (ML) model and using this trained model to predict the objective function value of molecules for which the objective function has not yet been calculated. Three surrogate model architectures were investigated in these studies: random forest (RF), feedforward neural network (NN), and directed message passing neural network (MPN) models.

#### Random forest models

Random forest (RF) models operate by ensembling decision tree models each trained on a random subset of the training data. Broadly, a decision tree is trained on input data of the form (***x***, *y*) where ***x*** = [*x*_1_, …, *x*_*N*_] is a vector composed of input features *x*_1_, …, *x*_*N*_ and *y* is a “target” output value. During training, a decision tree is built by progressively partitioning the input space at decision nodes into two subspaces corresponding to the value of a given feature. This process is repeated recursively on each of the resulting subspaces until some maximum amount of partitioning is achieved and a leaf node is constructed that contains all possible values {*y*_*i*_}_*i*=1_^*M*^ that correspond to the partitioning of the parent decision nodes. A more in-depth discussion on RF models for QSAR may be found in ref. 53. The RF surrogate model in our study was implemented using the RandomForestRegressor class from Scikit-Learn^54^ using an n_estimators value of 100 and a max_trees value of 8.

#### Feedforward neural networks

Feedforward neural networks (FFNNs) comprise an input layer, an output layer, and zero or more hidden layers between the two. At each layer, the input or hidden vector is linearly transformed by a learned weight matrix and passed through an elementwise nonlinear activation function. FFNNs are generally trained through stochastic gradient descent to minimize a loss function, which for regression tasks is generally the mean squared error.

The NN models in our study were implemented in TensorFlow^55^ using two fully connected hidden layers of 100 nodes each and an output size of one. Each hidden layer utilized a rectified linear unit (ReLU) activation function. The network was trained over 50 epochs using early stopping (patience = 5), an Adam optimizer with an initial learning rate of 0.01, a mean-squared error loss function with *L*2 regularization (0.01), and a batch size of 4096. Prediction uncertainties, as needed for non-greedy acquisition metrics, were estimated using Monte-Carlo Dropout *via* dropout layers (*p* = 0.2) after each hidden layer using 10 forward passes during inference.

#### Molecular fingerprints

Given that molecules are not naturally represented as feature vectors, inputs to both RF and NN models were generated by calculating molecular fingerprints. Fingerprint calculation algorithms vary in their implementation but can broadly be understood as encoding information about the presence or absence of substructures of the given molecule into a vector of fixed length. The input to both the RF and NN models used in this study is a 2048-bit atom-pair fingerprint^56^ with a minimum radius of one and a maximum radius of three. A more detailed overview of molecular fingerprints is provided in ref. 57.

#### Message passing neural networks

The third and final model architecture we tested was a directed message passing neural network (D-MPNN) model,^39^ a variant of a message passing neural network (MPNN) model. In contrast to FFNNs, MPNNs operate directly on the molecular graph rather than a fixed feature vector calculated from the graph. MPNNs function in two stages, an initial message passing phase followed by a readout phase. In the message passing phase, “messages” are passed between atoms and/or bonds and their direct neighbors and incoming messages are used to update the “hidden state” of each atom and/or bond. The message passing phase is repeated over multiple (*e.g.*, 3) iterations, at which point the hidden states of each atom are aggregated (*e.g.*, summed) to produce a molecule-level feature vector. By training this model at the same time as a FFNN operating on the feature vector, MPNNs are able to learn a task-specific representation of an input molecular graph. For more details on the D-MPNN model, we refer a reader to ref. 39.

Message passing neural network models were implemented using PyTorch^58^*via* PyTorchLightning^59^ with the MoleculeModel class from the Chemprop library^39^ using standard settings: messages passed on directed bonds, messages subjected to ReLU activation, a learned encoded representation of dimension 300, and the output of the message passing phase fully connected to an output layer of size 1. The model was trained using the Adam optimization algorithm, a Noam learning rate scheduler (initial, maximum, and final learning rates of 10^−4^, 10^−3^, and 10^−4^, respectively) and a root mean-squared error loss function over 50 epochs with a batch size of 50. For more details on the Noam learning rate scheduler, see ref. 60. The model was trained with early stopping tracking the validation score using a patience value of 10. When uncertainty values were needed for metric function calculation, an MVE model based off of the work done by Hirschfeld *et al.*^46^ was used. This model featured an output size of two and was trained using the loss function defined by Nix and Weigend:^61^2
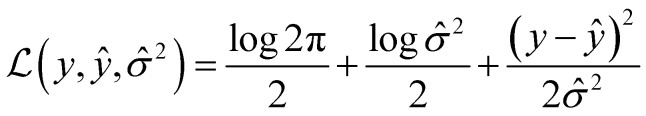


All of the surrogate models were used exactly as described above without additional hyperparameter optimization. The models were fully retrained from scratch with all acquired data at the beginning of each iteration.

### Datasets

The 10k, 50k, and HTS datasets used for these studies were generated from docking the compounds contained in both Discovery Diversity sets and the HTS collection from Enamine against thymidylate kinase (PDB ID: 4UNN). The docking was performed using AutoDock Vina with the following command line arguments:
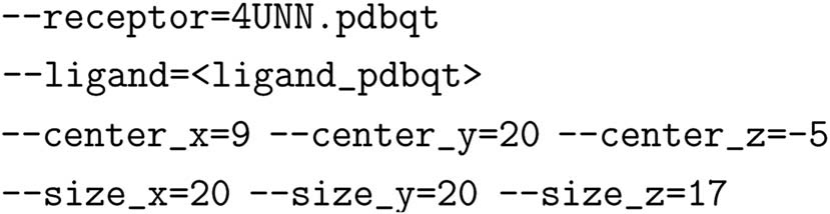


All other default arguments were left as-is. The docking score used in the datasets corresponds to the top output docking score. The ligands were prepared from SDFs available from Enamine,^62,63^ parsed into SMILES strings using RDKit,^64^ and processed into PDBQT files using OpenBabel^65^ with the –gen-3d flag. The receptor was prepared with PDBFixer^66^ using the PDB ID 4UNN, selecting only chain A, deleting all heterogens, adding all suggested missing residues and heavy atoms, and adding hydrogens for pH 7.0. Ligands that failed to dock were still included in the dataset without a corresponding score. MolPAL was still able to select these molecules during batch acquisition, but no objective function values for these points were returned and these points were not used in surrogate model training. The AmpC and D_4_ docking datasets are the publicly available datasets published by Lyu *et al.* and were used as provided.^40,41^ The AmpC Glide dataset was obtained from Yang *et al. via* email correspondence. We removed 499 docking scores with placeholder values of 10 000 kcal mol^−1^ (the remainder of the scores range between approximately −10 and +30 kcal mol^−1^). The Harvard Clean Energy Project dataset is the publicly available dataset of 2.4M organic photovoltaic materials and computed electronic properties: power conversion efficiency, HOMO, LUMO, *etc.*^67^ The score distribution of each dataset may be found in Fig. S6–S12.† The subsampled AmpC datasets were generated *via* the random selection of 2M datapoints from the full the AmpC dataset.

### Evaluation metrics

MolPAL performance was judged through three evaluation metrics: (1) average top-*k* docking score identified (“Average”), (2) the fraction of top-*k* SMILES identified (“SMILES”), and (3) the fraction of top-*k* scores identified (“Scores”). For (1), the average of the top-*k* scores of molecules explored by MolPAL was taken and divided by the true top-*k* molecules' scores based on the full dataset. (2) Was calculated by taking the intersection of the set of SMILES strings in the true top-*k* molecules and the found top-*k* molecules and dividing its size by *k*. (3) Was calculated as the size of the intersection of the list of true top-*k* scores and the list of observed top-*k* scores divided by *k*.

### Hyperparameter optimization

The experiments shown in this study represent only a small fraction of the configurations in which MolPAL may be run. A sample of settings that are supported include: various fingerprint types (*e.g.*, RDKit, Morgan, and MACCS), input preclustering for cluster-based acquisition, different confidence estimation methods for deep learning models, *etc.* Given the wealth of options, an exhaustive hyperparameter optimization was outside the scope of these investigations. We looked at broad trends in both the Enamine 10k and 50k datasets and found only minor variations in performance, supporting our choice not to pursue a rigorous screening of all possible configurations.

### Software design

MolPAL is built around the Explorer class, which performs the Bayesian optimization routine shown in Algorithm 1. The Explorer is designed with abstraction at its core and thus relies on four helper classes that each handles an isolated element of Bayesian optimization: MoleculePool, Model, Acquirer, and Objective. In each iteration of the Explorer's main optimization loop, a Model is first retrained on all observed data then applied to all molecules in the MoleculePool to generate a predicted mean and, depending on the Model, a predicted uncertainty for each molecule in the pool. These predictions are then passed to an Acquirer which calculates the acquisition utility of each molecule and acquires the top-*m* untested molecules from the MoleculePool based on their acquisition utilities. Next, this set of candidate molecules is handed to an Objective and the objective function value for each of these candidate molecules is calculated. Lastly, the stopping condition for the Explorer is checked and, if satisfied, the Explorer terminates and outputs the top-*k* evaluated molecules. A schematic of both the design and workflow of MolPAL may be seen in Fig. S1.† The experiments performed in this study were all performed retrospectively using the LookupObjective subclass of the Objective with a fully generated dataset as a lookup table for objective function calculation.

## Author contributions

D. E. G. wrote the MolPAL software and conducted the experiments; D. E. G. and C. W. C. designed the experiments; D. E. G., E. I. S., and C. W. C. wrote the manuscript; E. I. S. and C. W. C. supervised the work.

## Conflicts of interest

There are no conflicts to declare.

## Supplementary Material

SC-012-D0SC06805E-s001
