# Supplementary material for: Accelerating high-throughput virtual screening through molecular pool-based active learning
Source: Chem Sci. 2021 Apr 29;12(22):7866–81. doi: 10.1039/d0sc06805e (PMC8188596; doi:10.1039/d0sc06805e)
Supplement: SC-012-D0SC06805E-s001 [file SC-012-D0SC06805E-s001.pdf]

Electronic Supplementary Material (ESI) for Chemical Science.
This journal is © The Royal Society of Chemistry 2021

Supporting Information

Accelerating High-Throughput Virtual Screening Through Molecular Pool-Based Active Learning

David E. Graff,† Eugene Shakhnovich,† Connor W. Coley*,‡

†*Department of Chemistry and Chemical Biology, Harvard University, Cambridge, MA*

‡*Department of Chemical Engineering, MIT, Cambridge, MA*

E-mail: ccoley@mit.edu

Additional Methods

Elaboration on software design

The design choices detailed in the Software design paragraph of the Methods section are critical to both the testing and extension of the `MolPAL` software. Namely, the decision to rely on the `MoleculePool`, `Model`, `Acquirer`, and `Objective` helper classes enables the rapid and facile testing of different combinations of model architectures and acquisition strategies for a given objective optimization. This choice also enables the straightforward extension of `MolPAL` with new surrogate model architectures, acquisition metrics, and objective functions. The `Model` and `Objective` are both defined as minimal abstract base classes built around an adapter design pattern. This enables the simple interfacing of popular machine learning learning libraries (e.g., Scikit-Learn, PyTorch, and TensorFlow) via the `Model` and virtual screening software via the `Objective` with the `Explorer` class. The `MoleculePool` is primarily an abstraction of a list of molecules stored. This class stores both a molecule's SMILES string and, if necessary, its precalculated fingerprint. The fingerprint is used for

clustering, if desired, and as input to models expecting vectors as inputs (e.g., RF and NN models) during the model inference step. The data stored by the `MoleculePool` is all stored on disk to enable the seamless application of `MolPAL` to all-sizes of virtual libraries. Molecular graphs, the input to the MPN model, are not capable of being stored either in memory or on disk due to their large memory footprint in their current implementation. As such, they are recalculated as necessary.

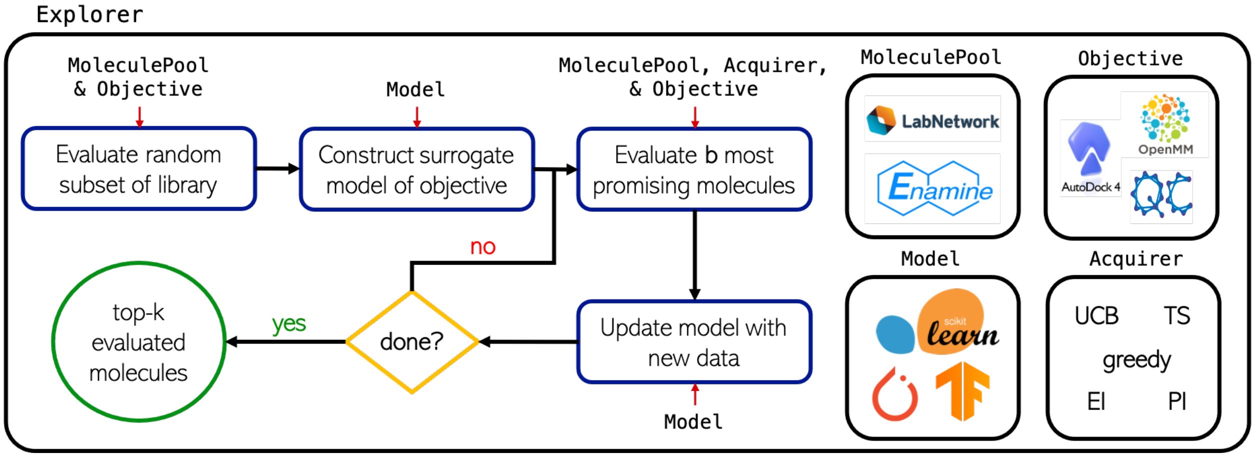

Figure S1: Overview of the MolPAL software structure and workflow.

Alternative surrogate models

Feedforward neural network models Two alternative NN models were defined for confidence estimation purposes: an ensemble model and a mean-variance estimation (MVE) model. The ensemble model was the same as the base model, with the only difference being that an ensemble of five models was trained. Each of the trained models was used for inference, and these five separate predictions were averaged and a variance taken to produce both a mean predicted value and an uncertainty estimate, respectively. The mean-variance estimation model used an output layer size of two, the learning rate was increased to 0.05 from 0.01, and the same loss function from the MPN-MVE was used (Equation 2). Neither of these alternate models was used for experiments due to their lower performance as compared to the dropout model.

Directed-message passing neural network models An MPN dropout model was also defined for confidence estimation purposes. This model was built similar to the NN dropout model, with the key difference being that the dropout layer was prepended to the hidden layer. Again, a dropout probability of 0.2 was used and dropout was performed during model inference. Mean predicted values were calculated by averaging 10 forward passes through the model and the variance of these predictions was used to as the predicted uncertainty. This alternate model was not used in experiments due to its significantly higher inference costs.

Retraining strategy

In addition to fully retraining the surrogate model from scratch using all acquired data, we tested an online training strategy. For online training, the trained surrogate model from the previous epoch was trained only on newly acquired data. Note that online training applies only to the NN and MPN models, as the RF is reinitialized each time it is fit.

Additional Results

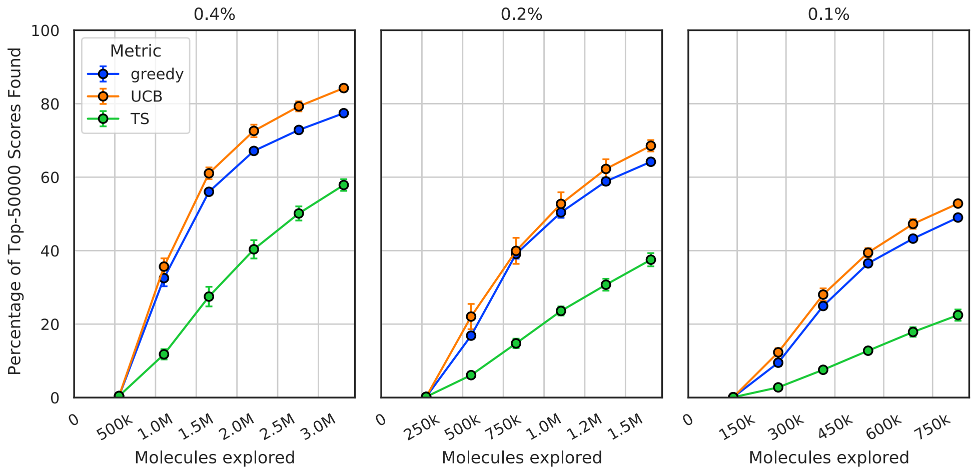

Figure S2: Bayesian optimization performance on the D_4 docking data (138M) as measured by the percentage of top-50000 scores found as a function of the number of ligands evaluated. Each trace represents the performance of the given acquisition metric using an MPN surrogate model. Chart labels represent the fraction of the fraction of the library taken in both the initialization batch and the five exploration batches. Error bars reflect ± one standard deviation across three runs.

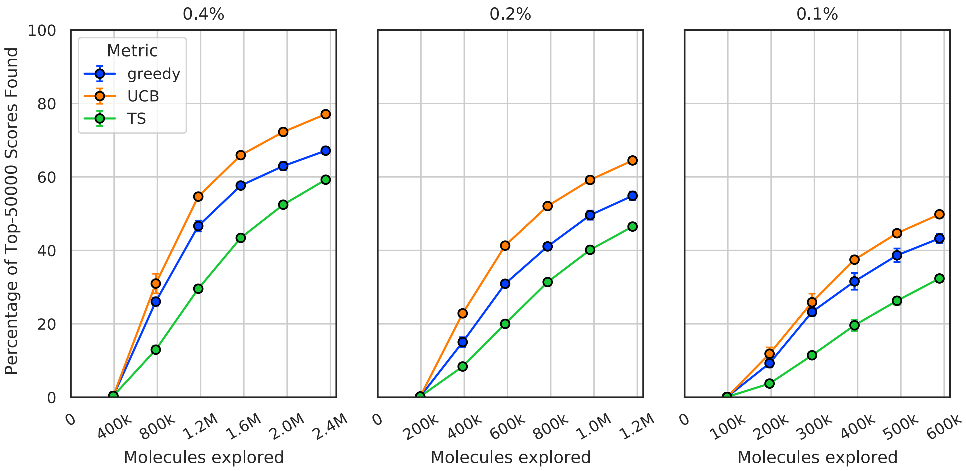

Figure S3: Bayesian optimization performance on the AmpC Glide docking data (98.2M) as measured by the percentage of top-50000 scores found as a function of the number of ligands evaluated. Each trace represents the performance of the given acquisition metric using an MPN surrogate model. Chart labels represent the fraction of the fraction of the library taken in both the initialization batch and the five exploration batches. Error bars reflect ± one standard deviation across three runs.

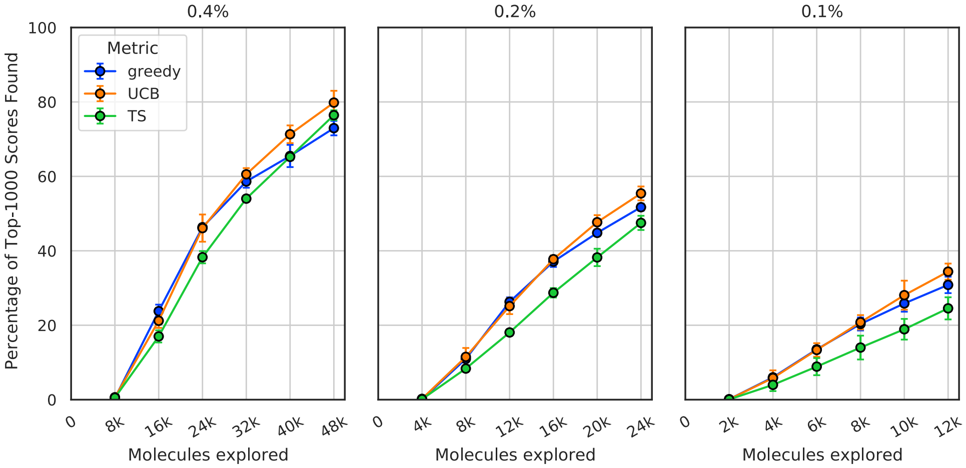

Figure S4: Bayesian optimization performance on random 2M subsets of the full AmpC docking data as measured by the percentage of top-1000 scores found as a function of the number of ligands evaluated. Subsets were generated by randomly selecting 2M SMILES strings and their associated docking scores from the full AmpC dataset. Each trace represents the average performance of an MPN surrogate model with the given acquisition metric using across five independent subsets. Chart labels represent the fraction of the subset taken in both the initialization batch and the five exploration batches. Error bars reflect ± one standard deviation across five independent subsets.

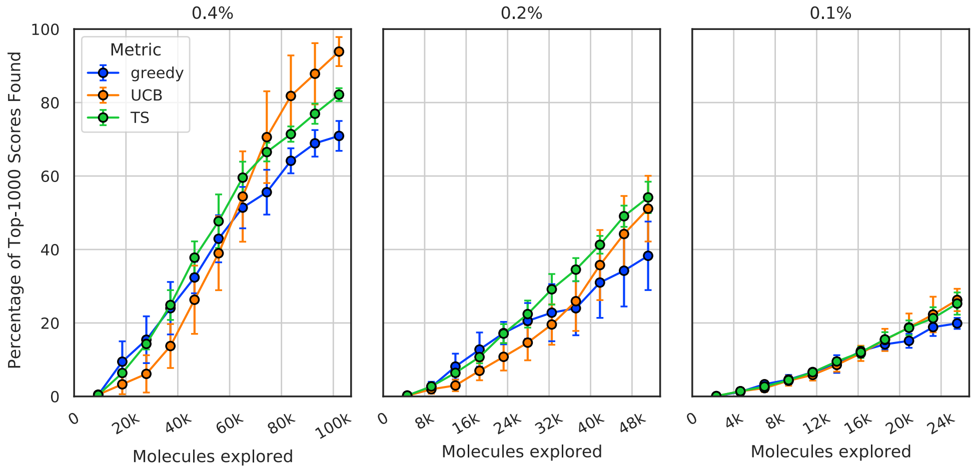

Figure S5: Bayesian optimization performance on the Harvard Clean Energy Project PCE data (2.4M) as measured by the percentage of top-1000 PCEs found as a function of the number of molecules evaluated. Each trace represents the performance of the given acquisition metric using an MPN surrogate model. Chart labels represent the fraction of the fraction of the library taken in both the initialization batch and the ten exploration batches. Error bars reflect ± one standard deviation across five runs.

Dataset score distributions

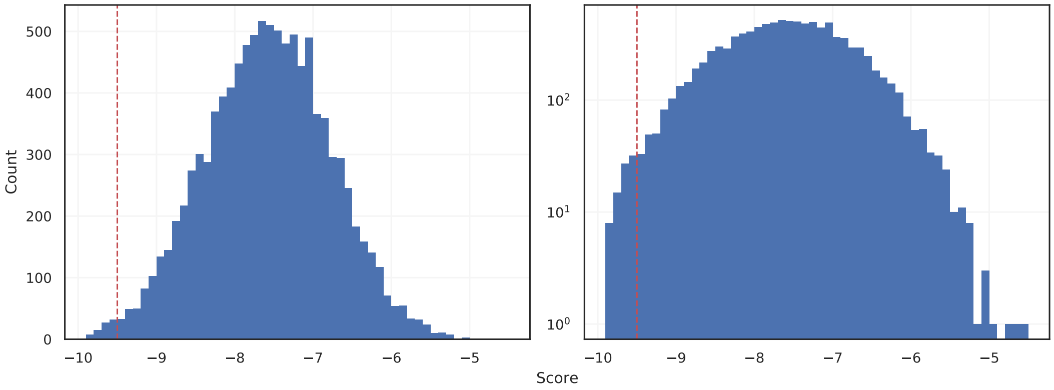

Figure S6: Distribution of docking scores in the Enamine 10k dataset with a bin size of 0.1. Red, dashed line corresponds to the kth best score ($k = 100$).

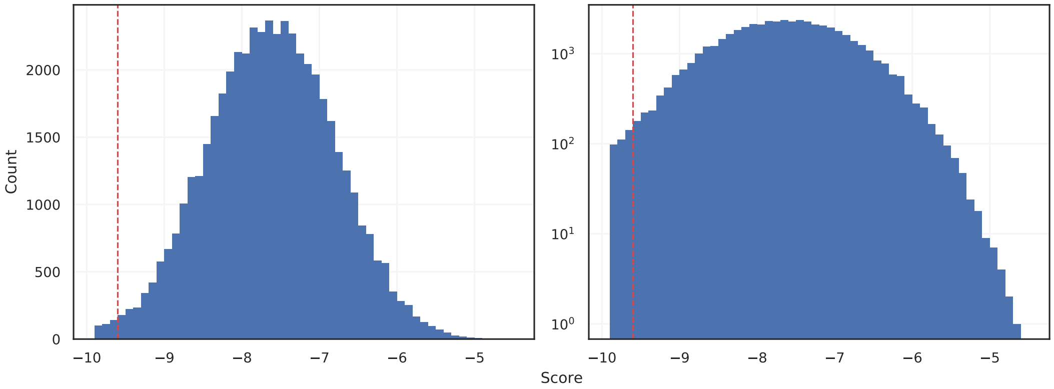

Figure S7: Distribution of docking scores in the Enamine 50k dataset with a bin size of 0.1. Red, dashed line corresponds to the kth best score ($k = 500$).

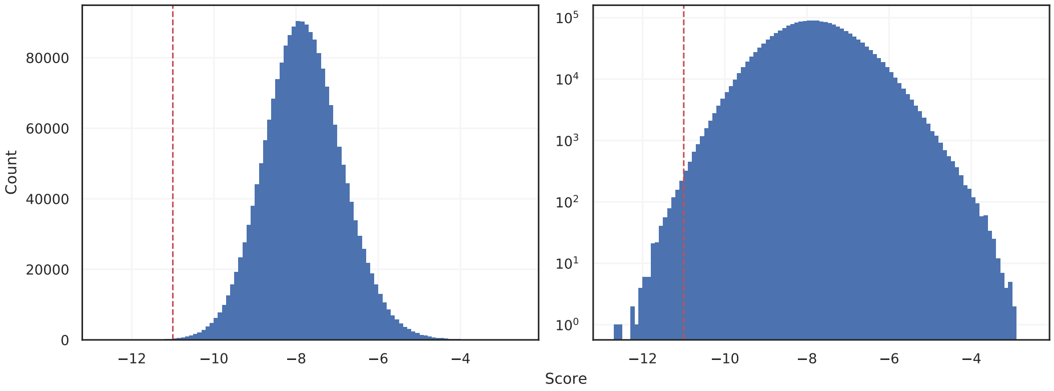

Figure S8: Distribution of docking scores in the Enamine HTS dataset (2.1M) with a bin size of 0.1. Red, dashed line corresponds to the kth best score ($k = 1000$).

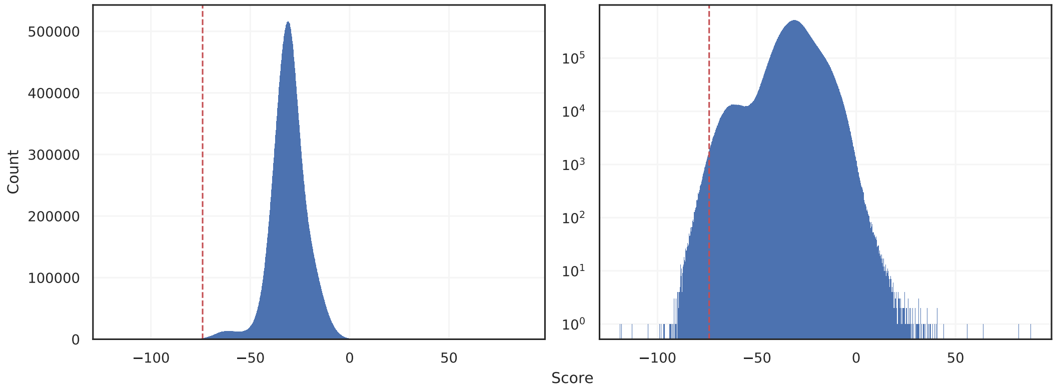

Figure S9: Distribution of docking scores in the AmpC dataset (99.5M) with a bin size of 0.1. Red, dashed line corresponds to the kth best score ($k = 50000$).

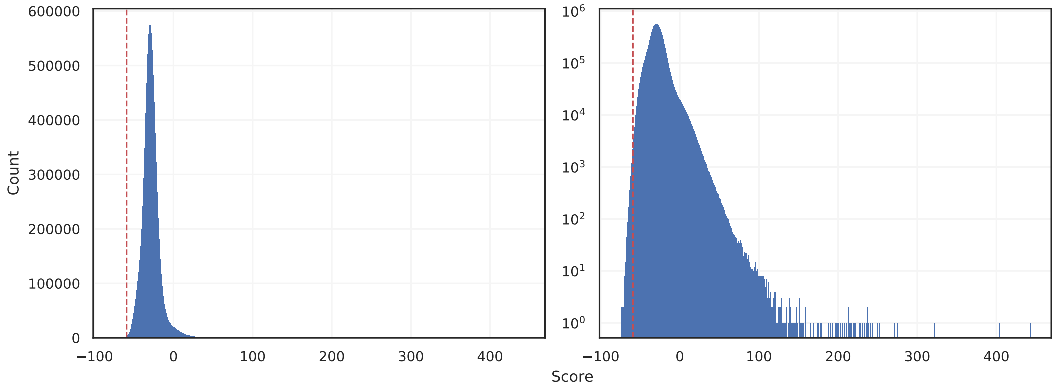

Figure S10: Distribution of docking scores in the D_4 dataset (138M) with a bin size of 0.1. Red, dashed line corresponds to the kth best score ($k = 50000$).

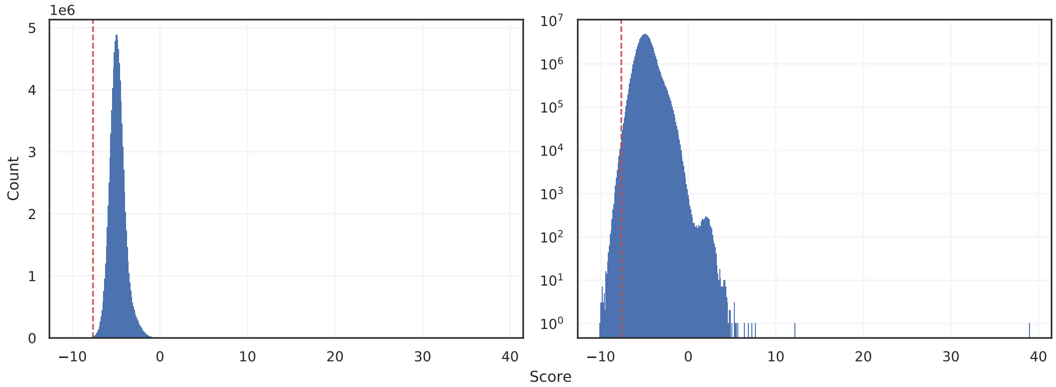

Figure S11: Distribution of docking scores in the AmpC Glide dataset (98.2M) with a bin size of 0.1. Red, dashed line corresponds to the kth best score ($k = 50000$).

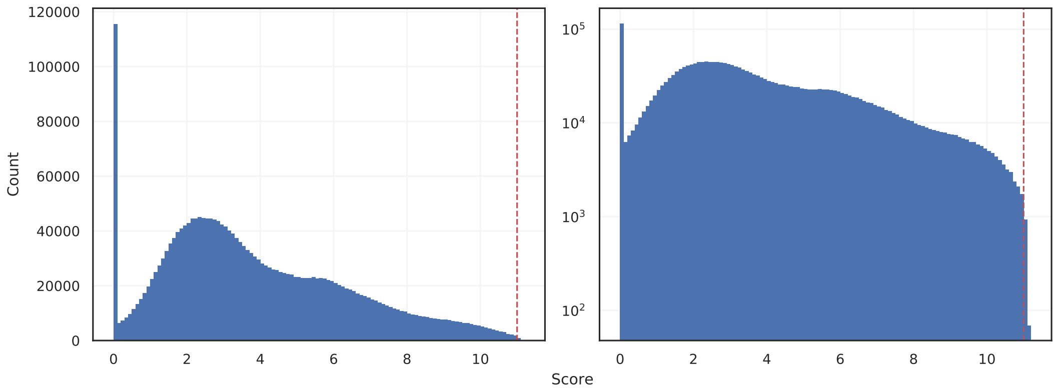

Figure S12: Distribution of docking scores in the HCEP dataset (2.4M) with a bin size of 0.1. Red, dashed line corresponds to the kth best score ($k = 1000$).

Library exploration across separate experiments

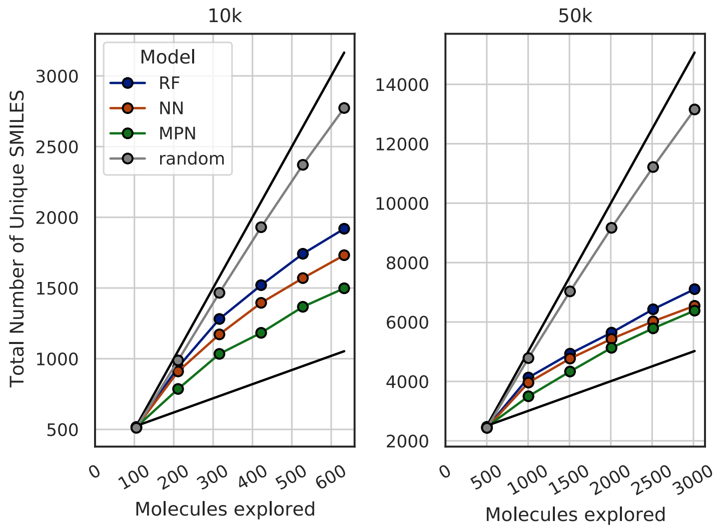

Figure S13: The total number of unique SMILES strings acquired across 5 greedy optimizations on the 10k and 50k libraries. The top black line is the theoretical maximum (i.e., repeated trials select distinct subsets of molecules to evaluate), and the bottom black line is the theoretical minimum (i.e., repeated trials select identical subsets of molecules to evaluate).

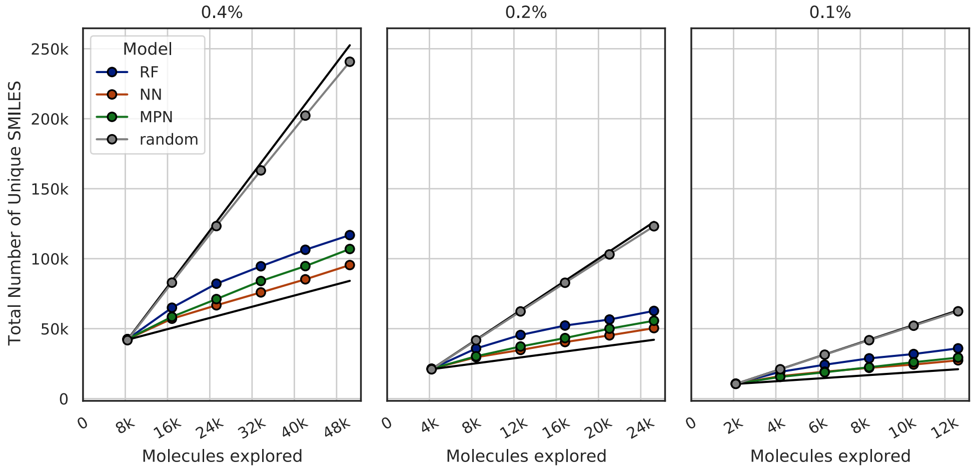

Figure S14: The total number of unique SMILES strings acquired across 5 greedy optimizations on the Enamine HTS docking dataset (2.1M). The top black line is the theoretical maximum (i.e., repeated trials select distinct subsets of molecules to evaluate), and the bottom black line is the theoretical minimum (i.e., repeated trials select identical subsets of molecules to evaluate). Chart labels represent the fraction of the fraction of the library taken in both the initialization batch and the five exploration batches.

Online training strategy

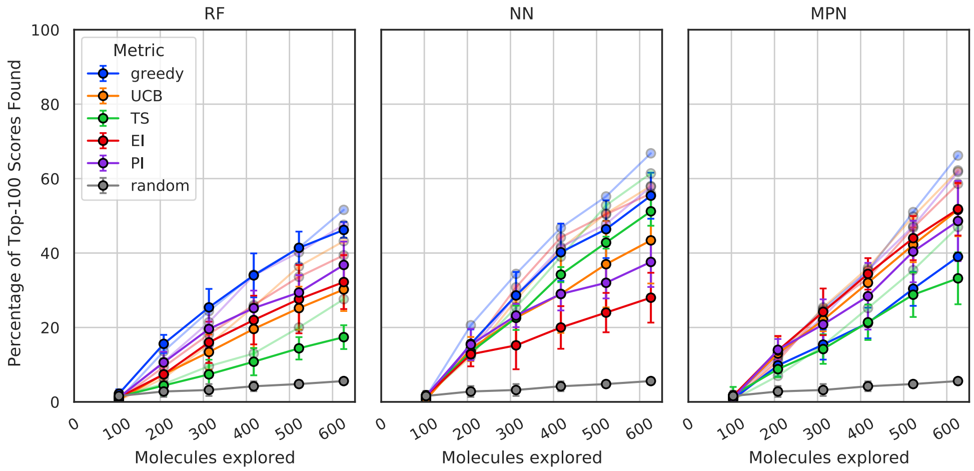

Figure S15: Bayesian optimization performance on Enamine 10k docking data as measured by the percentage of top-100 scores found as a function of the number of ligands evaluated. Each trace represents the performance of the given acquisition metric with the surrogate model architecture corresponding to the chart label. Full opacity: online model training. Faded: full model retraining. Each experiment began with a random 1% acquisition (ca. 100 samples) and acquired 1% more each iteration for five iterations. Error bars reflect ± one standard deviation across five runs.

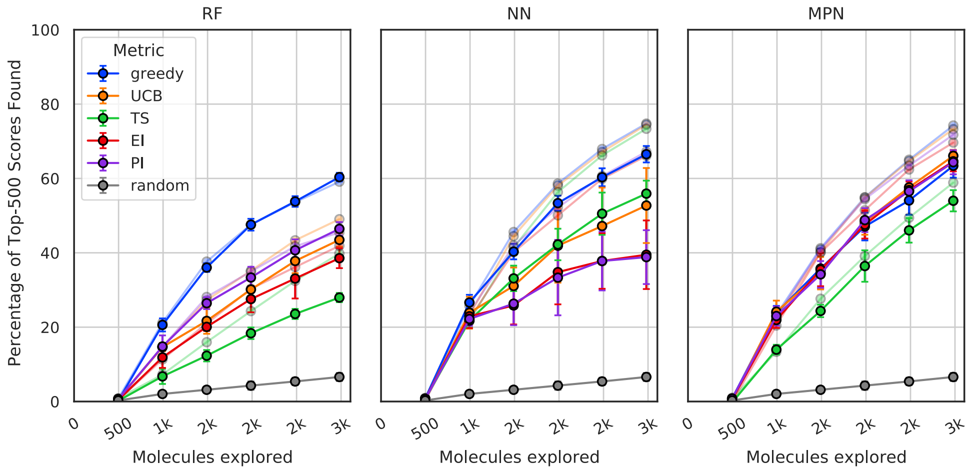

Figure S16: Bayesian optimization performance on Enamine 50k docking data as measured by the percentage of top-500 scores found as a function of the number of ligands evaluated. Each trace represents the performance of the given acquisition metric with the surrogate model architecture corresponding to the chart label. Full opacity: online model training. Faded: full model retraining. Each experiment began with a random 1% acquisition (ca. 500 samples) and acquired 1% more each iteration for five iterations. Error bars reflect ± one standard deviation across five runs.

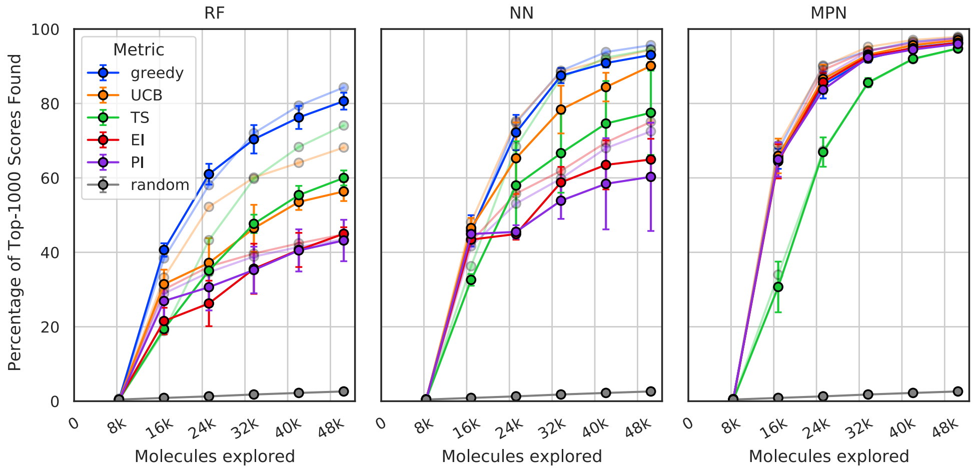

Figure S17: Bayesian optimization performance on Enamine HTS docking data (2.1M) as measured by the percentage of top-1000 scores found as a function of the number of ligands evaluated. Each trace represents the performance of the given acquisition metric with the surrogate model architecture corresponding to the chart label. Full opacity: online model training. Faded: full model retraining. Each experiment began with a random 0.4% acquisition (ca. 8,400 samples) and acquired 0.4% more each iteration for five iterations. Error bars reflect ± one standard deviation across five runs.

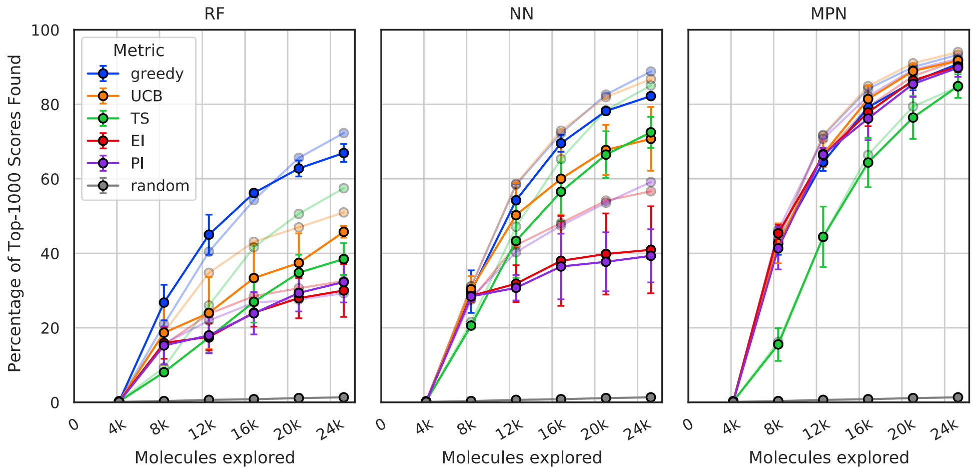

Figure S18: Bayesian optimization performance on Enamine HTS docking data (2.1M) as measured by the percentage of top-1000 scores found as function of the number of ligands evaluated. Each trace represents the performance of the given acquisition metric with the surrogate model architecture corresponding to the chart label. Full opacity: online model training. Faded: full model retraining. Each experiment began with a random 0.2% acquisition (ca. 4,200 samples) and acquired 0.2% more each iteration for five iterations. Error bars reflect ± one standard deviation across five runs.

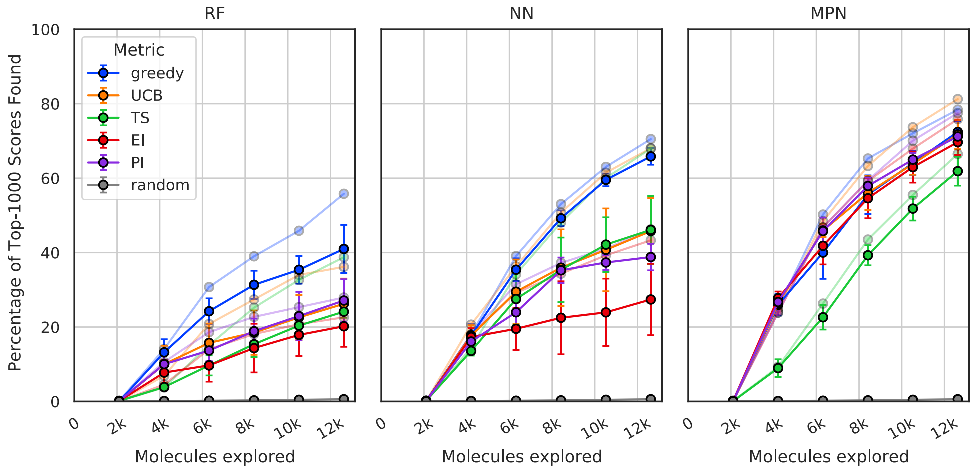

Figure S19: Bayesian optimization performance on Enamine HTS docking data (2.1M) as measured by the percentage of top-1000 scores found as function of the number of ligands evaluated. Each trace represents the performance of the given acquisition metric with the surrogate model architecture corresponding to the chart label. Full opacity: online model training. Faded: full model retraining. Each experiment began with a random 0.1% acquisition (ca. 2,100 samples) and acquired 0.1% more each iteration for five iterations. Error bars reflect ± one standard deviation across five runs.

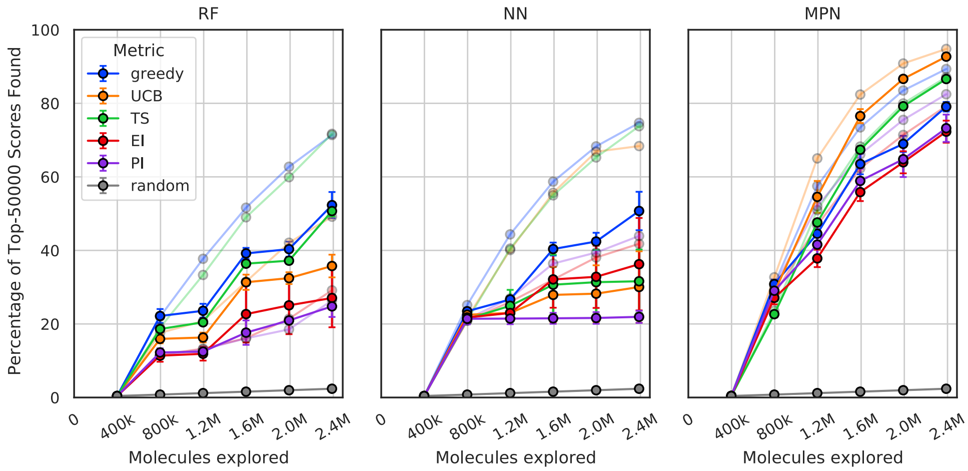

Figure S20: Bayesian optimization performance on AmpC docking data (99.5M) as measured by the percentage of top-50000 scores found as a function of the number of ligands evaluated. Each trace represents the performance of the given acquisition metric with the surrogate model architecture corresponding to the chart label. Full opacity: online model training. Faded: full model retraining. Each experiment began with a random 0.4% acquisition (ca. 40,000 samples) and acquired 0.4% more each iteration for five iterations. Error bars reflect ± one standard deviation across three runs.

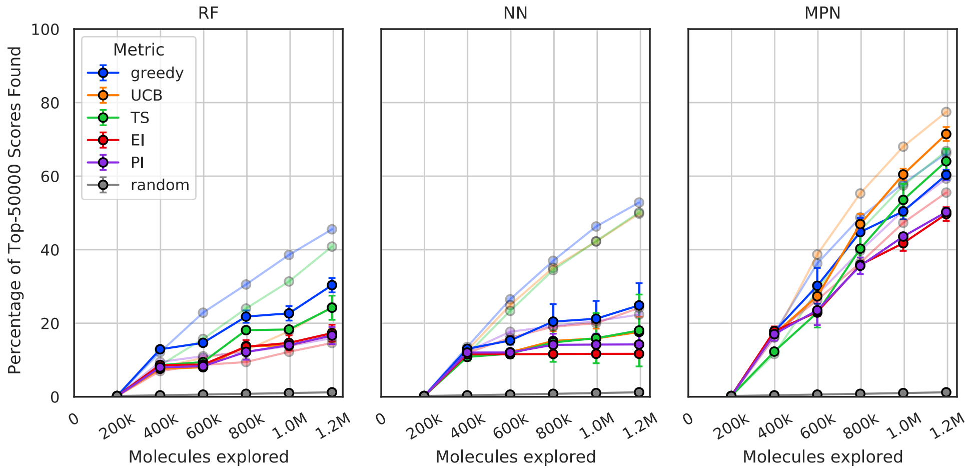

Figure S21: Bayesian optimization performance on AmpC docking data (99.5M) as measured by the percentage of top-50000 scores found as a function of the number of ligands evaluated. Each trace represents the performance of the given acquisition metric with the surrogate model architecture corresponding to the chart label. Full opacity: online model training. Faded: full model retraining. Each experiment began with a random 0.2% acquisition (ca. 20,000 samples) and acquired 0.2% more each iteration for five iterations. Error bars reflect ± one standard deviation across three runs.

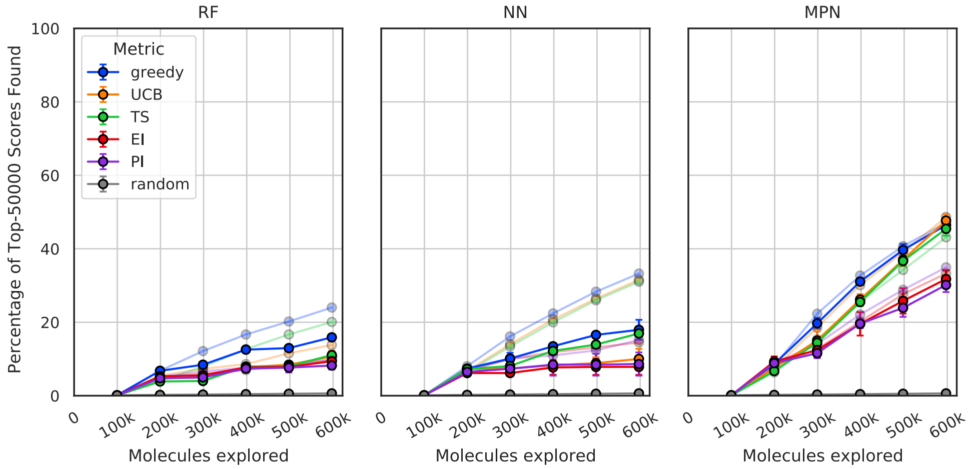

Figure S22: Bayesian optimization performance on AmpC docking data (99.5M) as measured by the percentage of top-50000 scores found as a function of the number of ligands evaluated. Each trace represents the performance of the given acquisition metric with the surrogate model architecture corresponding to the chart label. Full opacity: online model training. Faded: full model retraining. Each experiment began with a random 0.1% acquisition (ca. 10,000 samples) and acquired 0.1% more each iteration for five iterations. Error bars reflect ± one standard deviation across three runs.

Bayesian Optimization Performance

Table S1: Final Bayesian optimization performance on Enamine 10k docking data with a 1.0% batch size as measured by the given evaluation metric using the top-100 compounds found. Results are expressed as percentages and reflect the average (standard deviation) over five runs where higher is better.

Training	Model	Metric	Scores (± s.d.)	SMILES (± s.d.)	Average (± s.d.)
online	RF	greedy	46.2 (2.1)	40.8 (3.7)	97.86 (0.19)
		UCB	30.2 (5.8)	26.8 (5.3)	96.46 (0.51)
		TS	17.4 (3.2)	15.2 (3.2)	94.58 (0.31)
		EI	32.2 (7.2)	27.0 (5.8)	96.53 (0.49)
		PI	36.8 (6.3)	31.4 (5.4)	97.06 (0.58)
	NN	greedy	55.4 (6.2)	49.8 (6.4)	98.49 (0.31)
		UCB	43.4 (11.6)	38.4 (10.1)	97.59 (0.82)
		TS	51.2 (3.9)	45.8 (3.5)	98.16 (0.15)
		EI	28.0 (6.7)	24.6 (6.2)	96.09 (1.12)
		PI	37.6 (6.7)	33.0 (5.4)	96.93 (0.92)
	MPN	greedy	39.0 (5.7)	33.6 (4.5)	97.35 (0.59)
		UCB	51.6 (7.1)	43.8 (6.8)	98.05 (0.45)
		TS	33.2 (7.0)	27.8 (5.2)	96.67 (0.67)
		EI	51.8 (7.1)	44.2 (7.0)	98.12 (0.38)
		PI	48.6 (10.8)	41.2 (9.1)	97.86 (0.61)
retrain	RF	greedy	51.6 (5.9)	44.8 (5.8)	98.21 (0.31)
		UCB	43.2 (3.4)	37.2 (3.1)	97.58 (0.25)
		TS	27.6 (1.9)	22.6 (2.7)	95.97 (0.34)
		EI	39.4 (9.5)	33.8 (9.1)	97.16 (0.76)
		PI	47.6 (4.2)	41.4 (3.3)	97.82 (0.25)
	NN	greedy	66.8 (5.4)	59.2 (6.1)	98.97 (0.20)
		UCB	58.0 (3.5)	51.2 (3.4)	98.59 (0.16)
		TS	61.4 (3.9)	54.6 (3.4)	98.73 (0.19)
		EI	56.0 (7.5)	49.8 (6.9)	98.42 (0.42)
		PI	57.8 (2.4)	51.6 (2.3)	98.55 (0.15)
	MPN	greedy	66.2 (3.8)	57.8 (3.2)	98.88 (0.11)
		UCB	62.2 (5.8)	54.8 (4.6)	98.69 (0.27)
		TS	47.0 (3.8)	41.2 (2.7)	97.79 (0.23)
		EI	58.6 (9.9)	51.2 (8.8)	98.50 (0.52)
		PI	61.8 (3.9)	53.2 (3.7)	98.67 (0.18)
random			5.6 (0.8)	5.0 (0.9)	91.41 (0.34)

Table S2: Final Bayesian optimization performance on Enamine 50k docking data with a 1.0% batch size as measured by the given evaluation metric using the top-500 compounds found. Results are expressed as percentages and reflect the average (standard deviation) over five runs where higher is better.

Training	Model	Metric	Scores (± s.d.)	SMILES (± s.d.)	Average (± s.d.)
online	RF	greedy	60.4 (1.2)	56.4 (0.9)	98.75 (0.05)
		UCB	43.5 (1.8)	41.4 (1.9)	97.72 (0.15)
		TS	28.0 (1.2)	26.7 (1.3)	96.30 (0.11)
		EI	38.6 (2.7)	37.1 (2.7)	97.33 (0.25)
		PI	46.4 (1.8)	44.6 (1.4)	98.00 (0.13)
	NN	greedy	66.5 (2.2)	62.8 (2.0)	99.09 (0.10)
		UCB	52.7 (10.1)	49.9 (9.3)	98.29 (0.54)
		TS	55.9 (3.5)	52.6 (3.3)	98.56 (0.17)
		EI	39.5 (9.2)	37.1 (8.6)	97.10 (1.05)
		PI	38.8 (7.2)	36.7 (6.8)	97.25 (0.70)
	MPN	greedy	63.4 (3.2)	59.6 (2.9)	98.94 (0.14)
		UCB	66.1 (1.6)	61.9 (1.6)	99.07 (0.07)
		TS	54.0 (2.9)	51.1 (2.7)	98.51 (0.16)
		EI	64.6 (2.7)	60.6 (2.4)	99.01 (0.09)
		PI	64.4 (3.3)	60.3 (3.2)	98.99 (0.12)
retrain	RF	greedy	59.1 (2.9)	55.1 (3.0)	98.74 (0.15)
		TS	39.8 (2.9)	37.6 (2.9)	97.49 (0.23)
		UCB	49.0 (1.4)	46.9 (1.3)	98.16 (0.11)
		EI	41.9 (2.7)	40.1 (2.7)	97.62 (0.19)
		PI	45.5 (2.4)	43.4 (2.2)	97.92 (0.15)
	NN	greedy	74.8 (1.1)	70.1 (1.1)	99.39 (0.05)
		UCB	74.4 (1.4)	70.0 (1.2)	99.38 (0.04)
		TS	73.4 (2.3)	68.9 (2.3)	99.35 (0.07)
	.	EI	66.1 (3.0)	62.2 (2.9)	99.08 (0.12)
		PI	67.2 (4.0)	63.1 (3.5)	99.08 (0.16)
	MPN	greedy	74.2 (1.0)	69.9 (1.0)	99.38 (0.03)
		UCB	73.3 (0.5)	68.9 (0.5)	99.35 (0.03)
		TS	58.9 (1.3)	55.4 (1.4)	98.76 (0.08)
		EI	69.6 (1.3)	65.4 (1.2)	99.22 (0.05)
		PI	71.8 (1.6)	67.4 (1.8)	99.30 (0.06)
random			6.6 (1.0)	6.1 (1.2	91.36 (0.19)

Table S3: Final Bayesian optimization performance on Enamine HTS docking data (2.1M) with a 0.4% batch size as measured by the given evaluation metric using the top-1000 compounds found. Results are expressed as percentages and reflect the average (standard deviation) over five runs where higher is better.

Training	Model	Metric	Scores (± s.d.)	SMILES (± s.d.)	Average (± s.d.)
online	RF	greedy	80.6 (2.3)	76.5 (2.1)	99.45 (0.05)
		UCB	56.4 (2.6)	54.0 (2.4)	98.59 (0.14)
		TS	60.0 (2.0)	57.1 (1.8)	98.69 (0.07)
		EI	45.0 (1.8)	43.5 (1.9)	97.93 (0.10)
		PI	43.2 (5.6)	41.8 (5.3)	97.80 (0.41)
	NN	greedy	93.0 (0.8)	89.8 (1.0)	99.79 (0.03)
		UCB	90.1 (1.1)	86.1 (1.6)	99.69 (0.03)
		TS	77.5 (11.3)	73.4 (10.5)	99.28 (0.40)
		EI	64.9 (5.6)	61.5 (5.5)	98.87 (0.24)
		PI	60.3 (14.5)	57.0 (13.8)	98.58 (0.55)
	MPN	greedy	96.3 (0.3)	94.1 (0.4)	99.91 (0.01)
		UCB	97.0 (0.5)	94.5 (0.2)	99.93 (0.01)
		TS	94.8 (0.7)	92.3 (1.2)	99.86 (0.03)
		EI	96.3 (0.4)	94.3 (0.2)	99.91 (0.01)
		PI	96.0 (0.8)	94.1 (0.8)	99.90 (0.02)
retrain	RF	greedy	84.3 (1.1)	79.8 (0.9)	99.53 (0.02)
		UCB	68.2 (2.7)	65.2 (2.6)	99.03 (0.10)
		TS	74.1 (1.0)	70.3 (1.2)	99.26 (0.04)
		EI	44.8 (4.0)	43.2 (3.9)	97.90 (0.26)
		PI	43.5 (2.6)	42.2 (2.7)	97.80 (0.18)
	NN	greedy	95.7 (0.1)	93.2 (0.1)	99.89 (0.00)
		UCB	94.4 (0.5)	91.5 (0.8)	99.84 (0.02)
		TS	94.5 (0.3)	91.7 (0.5)	99.84 (0.01)
		EI	75.1 (3.9)	71.2 (3.9)	99.26 (0.14)
		PI	72.5 (2.1)	69.0 (1.9)	99.19 (0.08)
	MPN	greedy	97.6 (0.3)	94.8 (0.1)	99.94 (0.01)
		UCB	97.9 (0.6)	94.8 (0.3)	99.95 (0.01)
		TS	94.7 (1.1)	92.2 (1.5)	99.84 (0.03)
		EI	97.4 (0.2)	94.8 (0.1)	99.94 (0.00)
		PI	97.6 (0.5)	94.8 (0.2)	99.94 (0.01)
random			2.6 (0.1)	2.4 (0.1)	90.09 (0.15)

Table S4: Final Bayesian optimization performance on Enamine HTS docking data (2.1M) with a 0.2% batch size as measured by the given evaluation metric using the top-1000 compounds found. Results are expressed as percentages and reflect the average (standard deviation) over five runs where higher is better.

Training	Model	Metric	Scores (± s.d.)	SMILES (± s.d.)	Average (± s.d.)
online	RF	greedy	66.9 (2.4)	64.0 (2.4)	99.01 (0.11)
		UCB	45.8 (1.6)	44.1 (1.4)	97.94 (0.09)
		TS	38.5 (4.3)	36.9 (4.0)	97.39 (0.31)
		EI	30.0 (7.1)	29.0 (7.1)	96.46 (0.75)
		PI	32.3 (5.5)	31.1 (5.2)	96.79 (0.60)
	NN	greedy	82.2 (0.8)	78.1 (0.6)	99.47 (0.03)
		UCB	70.7 (8.6)	67.9 (8.3)	99.13 (0.38)
		TS	72.5 (4.2)	68.9 (3.9)	99.19 (0.14)
		EI	40.9 (11.7)	38.9 (11.1)	97.49 (0.80)
		PI	39.3 (7.1)	37.4 (6.9)	97.46 (0.48)
	MPN	greedy	90.8 (1.7)	86.5 (1.8)	99.70 (0.05)
		UCB	91.8 (0.8)	88.0 (1.1)	99.74 (0.03)
		TS	84.9 (3.2)	80.6 (2.9)	99.54 (0.08)
		EI	90.2 (1.6)	86.2 (2.0)	99.70 (0.05)
		PI	89.8 (2.5)	85.9 (2.4)	99.70 (0.06)
retrain	RF	greedy	72.3 (1.9)	69.0 (1.9)	99.23 (0.08)
		UCB	51.0 (2.9)	48.9 (2.9)	98.25 (0.15)
		TS	57.5 (1.4)	54.8 (1.5)	98.60 (0.05)
		EI	32.6 (3.1)	31.3 (3.0)	96.87 (0.28)
		PI	29.3 (5.1)	28.3 (5.0)	96.54 (0.52)
	NN	greedy	88.8 (0.8)	83.9 (0.8)	99.63 (0.03)
		UCB	86.7 (0.5)	82.1 (0.6)	99.59 (0.01)
		TS	85.0 (0.9)	80.4 (0.9)	99.53 (0.03)
		EI	56.6 (4.3)	54.0 (4.1)	98.54 (0.23)
		PI	59.1 (3.1)	56.6 (3.0)	98.67 (0.13)
	MPN	greedy	93.3 (0.9)	89.8 (1.0)	99.80 (0.04)
		UCB	94.0 (0.4)	91.0 (0.8)	99.83 (0.01)
		TS	84.7 (1.5)	80.2 (1.3)	99.53 (0.05)
		EI	91.8 (1.1)	87.8 (1.3)	99.76 (0.03)
		PI	92.3 (0.5)	88.4 (0.5)	99.77 (0.01)
random			1.3 (0.4)	1.3 (0.3)	87.75 (0.14)

Table S5: Final Bayesian optimization performance on Enamine HTS docking data (2.1M) with a 0.1% batch size as measured by the given evaluation metric using the top-1000 compounds found. Results are expressed as percentages and reflect the average (standard deviation) over five runs where higher is better.

Training	Model	Metric	Scores (± s.d.)	SMILES (± s.d.)	Average (± s.d.)
online	RF	greedy	41.0 (6.5)	39.5 (6.2)	97.60 (0.49)
		UCB	26.2 (6.8)	25.4 (6.6)	95.84 (0.91)
		TS	24.1 (1.6)	23.1 (1.5)	95.63 (0.33)
		EI	20.2 (5.5)	19.6 (5.2)	94.90 (1.29)
		PI	27.1 (5.7)	26.4 (5.6)	96.05 (0.85)
	NN	greedy	65.8 (2.2)	63.2 (2.0)	98.96 (0.09)
		UCB	45.8 (8.9)	43.9 (8.6)	97.90 (0.62)
		TS	46.1 (9.1)	44.3 (8.8)	97.90 (0.62)
		EI	27.4 (9.6)	26.2 (9.4)	96.22 (0.98)
		PI	38.8 (3.6)	37.3 (3.3)	97.39 (0.30)
	MPN	greedy	72.4 (2.8)	69.2 (2.5)	99.21 (0.08)
		UCB	71.7 (3.9)	68.6 (3.5)	99.20 (0.12)
		TS	61.9 (3.9)	58.9 (3.7)	98.79 (0.16)
		EI	69.7 (3.5)	66.8 (3.2)	99.16 (0.13)
		PI	71.2 (1.3)	68.2 (1.0)	99.21 (0.07)
retrain	RF	greedy	55.8 (4.9)	53.4 (4.6)	98.54 (0.24)
		UCB	36.2 (4.2)	34.9 (4.2)	97.16 (0.42)
		TS	38.8 (2.5)	37.1 (2.6)	97.43 (0.18)
		EI	22.6 (2.7)	21.9 (2.6)	95.62 (0.37)
		PI	27.9 (2.7)	27.1 (2.7)	96.27 (0.37)
	NN	greedy	70.5 (1.8)	66.9 (1.6)	99.08 (0.09)
		UCB	68.0 (0.9)	64.8 (1.2)	99.04 (0.05)
		TS	68.0 (0.8)	64.8 (0.7)	99.05 (0.03)
		EI	43.3 (3.8)	41.5 (3.5)	97.74 (0.29)
		PI	46.3 (2.3)	44.2 (2.2)	97.94 (0.17)
	MPN	greedy	78.4 (2.2)	74.4 (2.2)	99.37 (0.07)
		UCB	81.2 (0.8)	77.4 (0.6)	99.46 (0.03)
		TS	66.5 (2.0)	63.3 (1.9)	98.98 (0.08)
		PI	77.5 (2.0)	74.2 (1.7)	99.40 (0.05)
		EI	75.9 (1.0)	72.7 (1.0)	99.38 (0.03)
random			0.6 (0.2)	0.5 (0.2)	85.36 (0.11)

Table S6: Final Bayesian optimization performance on AmpC docking data (99.5M) with a 0.4% batch size as measured by the given evaluation metric using the top-50000 compounds found. Results are expressed as percentages and reflect the average (standard deviation) over three runs where higher is better.

Training	Model	Metric	Scores (± s.d.)	SMILES (± s.d.)	Average (± s.d.)
online	RF	greedy	52.3 (3.6)	52.3 (3.6)	97.70 (0.25)
		UCB	35.8 (3.1)	35.7 (3.1)	96.29 (0.32)
		TS	50.7 (1.5)	50.7 (1.5)	97.59 (0.11)
		EI	27.1 (8.0)	27.0 (8.0)	95.04 (1.12)
		PI	24.8 (2.9)	24.8 (2.9)	94.81 (0.51)
	NN	greedy	50.7 (5.2)	50.7 (5.2)	97.59 (0.38)
		UCB	30.0 (9.7)	30.0 (9.7)	95.41 (1.16)
		TS	31.6 (8.6)	31.6 (8.6)	95.64 (1.11)
		EI	36.3 (12.6)	36.2 (12.6)	96.06 (1.38)
		PI	21.9 (1.6)	21.9 (1.6)	94.34 (0.30)
	MPN	greedy	79.1 (1.3)	79.0 (1.3)	99.19 (0.06)
		UCB	92.7 (0.6)	92.7 (0.6)	99.78 (0.02)
		TS	86.6 (0.2)	86.6 (0.2)	99.52 (0.01)
		EI	72.3 (3.0)	72.2 (3.0)	99.04 (0.15)
		PI	73.2 (3.7)	73.2 (3.7)	99.08 (0.18)
retrain	RF .	greedy	71.4 (2.1)	71.3 (2.1)	98.79 (0.13)
		UCB	49.2 (7.7)	49.1 (7.7)	97.46 (0.58)
		TS	71.7 (1.9)	71.6 (1.9)	98.78 (0.10)
		EI	29.1 (4.4)	29.1 (4.4)	95.47 (0.59)
		PI	26.4 (4.7)	26.4 (4.7)	95.03 (0.71)
	NN	greedy	74.7 (1.4)	74.6 (1.4)	98.94 (0.08)
		UCB	68.4 (1.4)	68.3 (1.4)	98.65 (0.07)
		TS	73.8 (1.2)	73.7 (1.2)	98.92 (0.05)
		EI	41.8 (1.8)	41.8 (1.8)	96.90 (0.16)
		PI	43.9 (2.1)	43.9 (2.1)	97.07 (0.17)
	MPN	greedy	89.3 (0.2)	89.3 (0.2)	99.61 (0.01)
		UCB	94.8 (0.2)	94.8 (0.2)	99.83 (0.01)
		TS	87.1 (0.3)	87.1 (0.3)	99.54 (0.01)
		EI	79.2 (2.8)	79.2 (2.8)	99.34 (0.11)
		PI	82.5 (1.4)	82.4 (1.4)	99.47 (0.05)
random			2.4 (0.1)	2.4 (0.1)	81.03 (0.04)

Table S7: Final Bayesian optimization performance on AmpC docking data (99.5M) with a 0.2% batch size as measured by the given evaluation metric using the top-50000 compounds found. Results are expressed as percentages and reflect the average (standard deviation) over three runs where higher is better.

Training	Model	Metric	Scores (± s.d.)	SMILES (± s.d.)	Average (± s.d.)
online	RF	greedy	30.4 (2.0)	30.3 (2.0)	95.67 (0.24)
		UCB	16.8 (1.8)	16.8 (1.8)	93.23 (0.46)
		TS	24.2 (3.3)	24.2 (3.3)	94.75 (0.54)
		EI	17.3 (2.3)	17.3 (2.3)	93.03 (0.66)
		PI	16.7 (2.3)	16.7 (2.3)	92.96 (0.63)
	NN	greedy	24.9 (6.0)	24.8 (6.0)	94.69 (1.09)
		UCB	17.6 (4.2)	17.6 (4.2)	93.29 (1.14)
		TS	18.0 (9.8)	18.0 (9.8)	92.83 (2.12)
		EI	11.7 (0.4)	11.7 (0.4)	91.48 (0.22)
		PI	14.2 (3.0)	14.2 (3.0)	92.26 (0.88)
	MPN	greedy	60.3 (1.4)	60.3 (1.4)	98.23 (0.10)
		UCB	71.4 (1.9)	71.4 (1.9)	98.99 (0.10)
		TS	64.0 (3.4)	64.0 (3.4)	98.50 (0.18)
		EI	49.7 (1.9)	49.6 (1.9)	97.62 (0.15)
		PI	50.2 (1.1)	50.2 (1.1)	97.68 (0.09)
retrain	RF	greedy	45.5 (1.8)	45.5 (1.8)	97.19 (0.14)
		UCB	24.4 (2.0)	24.4 (2.0)	94.81 (0.40)
		TS	40.8 (1.9)	40.8 (1.9)	96.80 (0.17)
		EI	14.6 (2.7)	14.6 (2.7)	92.44 (0.85)
		PI	16.0 (1.6)	16.0 (1.6)	92.83 (0.44)
	NN	greedy	52.8 (0.5)	52.8 (0.5)	97.72 (0.03)
		UCB	49.8 (0.5)	49.8 (0.5)	97.52 (0.04)
		TS	50.1 (1.0)	50.1 (1.0)	97.53 (0.07)
		EI	24.2 (1.0)	24.2 (1.0)	94.75 (0.17)
		PI	22.3 (1.1)	22.3 (1.1)	94.42 (0.19)
	MPN	greedy	66.2 (1.2)	66.1 (1.2)	98.51 (0.06)
		UCB	77.5 (1.9)	77.4 (1.9)	99.25 (0.07)
		TS	66.8 (0.3)	66.8 (0.3)	98.65 (0.01)
		EI	55.5 (1.7)	55.5 (1.7)	98.09 (0.12)
		PI	59.3 (1.0)	59.3 (1.0)	98.35 (0.06)
random			1.2 (0.0)	1.2 (0.0)	72.23 (0.10)

Table S8: Final Bayesian optimization performance on AmpC docking data (99.5M) with a 0.1% batch size as measured by the given evaluation metric using the top-50000 compounds found. Results are expressed as percentages and reflect the average (standard deviation) over three runs where higher is better.

Training	Model	Metric	Scores (± s.d.)	SMILES (± s.d.)	Average (± s.d.)
online	RF	greedy	15.9 (0.4)	15.9 (0.4)	93.01 (0.09)
		UCB	10.7 (1.6)	10.7 (1.6)	90.49 (0.96)
		TS	11.1 (0.1)	11.0 (0.1)	91.25 (0.10)
		EI	9.4 (1.8)	9.4 (1.8)	89.75 (1.22)
		PI	8.2 (1.1)	8.2 (1.1)	89.24 (0.59)
	NN	greedy	17.9 (2.7)	17.9 (2.7)	93.28 (0.67)
		UCB	10.0 (2.7)	10.0 (2.7)	90.16 (1.78)
		TS	16.9 (0.9)	16.9 (0.9)	93.16 (0.24)
		EI	7.9 (2.2)	7.8 (2.2)	89.01 (1.39)
		PI	8.6 (3.2)	8.6 (3.2)	89.24 (1.87)
	MPN	greedy	46.5 (1.9)	46.4 (1.9)	97.25 (0.16)
		UCB	47.7 (1.7)	47.7 (1.7)	97.41 (0.17)
		TS	45.4 (1.9)	45.4 (1.9)	97.19 (0.17)
		EI	31.8 (2.4)	31.7 (2.4)	95.59 (0.32)
		PI	30.2 (1.9)	30.1 (1.9)	95.32 (0.37)
retrain	RF	greedy	24.0 (2.2)	24.0 (2.2)	94.76 (0.35)
		UCB	13.8 (1.0)	13.8 (1.0)	92.01 (0.42)
		TS	20.1 (2.0)	20.1 (2.0)	94.08 (0.38)
		EI	9.8 (2.3)	9.8 (2.3)	90.03 (1.03)
		PI	9.8 (1.3)	9.7 (1.3)	90.44 (0.54)
	NN	greedy	33.3 (0.3)	33.2 (0.3)	96.00 (0.03)
		UCB	31.5 (0.6)	31.5 (0.6)	95.80 (0.07)
		TS	31.0 (0.8)	31.0 (0.8)	95.73 (0.10)
		EI	14.5 (0.8)	14.5 (0.8)	92.41 (0.30)
		PI	15.2 (1.4)	15.2 (1.4)	92.72 (0.43)
	MPN	greedy	47.1 (1.8)	47.1 (1.8)	97.29 (0.15)
		UCB	48.7 (2.4)	48.6 (2.4)	97.50 (0.20)
		TS	43.1 (0.5)	43.1 (0.5)	96.99 (0.05)
		EI	33.3 (0.9)	33.2 (0.9)	95.82 (0.14)
		PI	34.9 (1.4)	34.9 (1.4)	96.05 (0.19)
random			0.6 (0.0)	0.6 (0.0)	64.44 (0.05)

Table S9: Final Bayesian optimization performance on D_4 docking data (138M) with an MPN surrogate model as measured by the given evaluation metric using the top-50000 compounds found. Batch size is expressed as a percentage of the total library size. Results are expressed as percentages and reflect the average (standard deviation) over three runs where higher is better.

Batch size	Metric	Scores (± s.d.)	SMILES (± s.d.)	Average (± s.d.)
0.4	greedy	77.4 (0.7)	77.3 (0.7)	99.27 (0.03)
	UCB	84.3 (0.9)	84.2 (0.9)	99.58 (0.03)
	TS	57.9 (1.6)	57.7 (1.6)	98.32 (0.09)
0.2	greedy	64.2 (0.9)	64.1 (0.9)	98.67 (0.05)
	UCB	68.6 (1.5)	68.5 (1.5)	98.96 (0.07)
	TS	37.5 (1.8)	37.4 (1.8)	96.65 (0.20)
0.1	greedy	49.0 (0.6)	48.9 (0.6)	97.70 (0.05)
	UCB	52.8 (0.5)	52.7 (0.5)	98.01 (0.04)
	TS	22.4 (1.5)	22.4 (1.5)	94.16 (0.34)

Table S10: Final Bayesian optimization performance on AmpC Glide docking data (98.2M) with an MPN surrogate model as measured by the given evaluation metric using the top-50000 compounds found. Batch size is expressed as a percentage of the total library size. Results are expressed as percentages and reflect the average (standard deviation) over three runs where higher is better.

Batch size	Metric	Scores (± s.d.)	SMILES (± s.d.)	Average (± s.d.)
0.4	greedy	70.2 (1.5)	70.2 (1.5)	99.00 (0.06)
	UCB	81.6 (0.1)	81.6 (0.1)	99.42 (0.00)
	TS	64.1 (1.1)	64.1 (1.1)	98.74 (0.05)
0.2	greedy	54.9 (1.1)	54.9 (1.1)	98.04 (0.07)
	UCB	64.5 (0.3)	64.5 (0.3)	98.65 (0.02)
	TS	46.5 (0.6)	46.5 (0.6)	97.42 (0.05)
0.1	greedy	43.3 (1.2)	43.3 (1.2)	97.14 (0.11)
	UCB	49.8 (0.3)	49.8 (0.3)	97.67 (0.04)
	TS	32.4 (0.9)	32.4 (0.9)	95.75 (0.14)

Table S11: Final Bayesian optimization performance on subsampled AmpC docking data (2M) with an MPN surrogate model as measured by the given evaluation metric using the top-1000 compounds found. Batch size is expressed as a percentage of the total library size. Results are expressed as percentages and reflect the average (standard deviation) over five independent subsets where higher is better.

Batch size	Metric	Scores (± s.d.)	SMILES (± s.d.)	Average (± s.d.)
0.4	greedy	73.0 (1.9)	72.9 (1.9)	98.88 (0.12)
	UCB	79.8 (3.2)	79.8 (3.2)	99.22 (0.18)
	TS	76.4 (1.3)	76.4 (1.3)	99.07 (0.10)
0.2	greedy	51.7 (1.0)	51.7 (1.0)	97.63 (0.13)
	UCB	55.4 (1.9)	55.4 (1.8)	97.93 (0.12)
	TS	47.5 (1.9)	47.5 (1.9)	97.37 (0.12)
0.1	greedy	30.8 (2.2)	30.8 (2.2)	95.73 (0.20)
	UCB	34.4 (2.2)	34.4 (2.2)	96.06 (0.27)
	TS	24.5 (3.0)	24.5 (3.0)	94.84 (0.34)

Table S12: Final Bayesian optimization performance on HCEP PCE data (2.4M) as measured by the given evaluation metric using the top-1000 compounds found. Batch size is expressed as a percentage of the total library size. Results are expressed as percentages and reflect the average (standard deviation) over five runs where higher is better.

Batch size	Model	Metric	Scores (± s.d.)	SMILES (± s.d.)	Average (± s.d.)
0.4	RF	greedy	13.2 (3.4)	13.2 (3.4)	98.38 (0.30)
		UCB	19.3 (2.0)	19.3 (2.0)	98.84 (0.10)
		TS	13.7 (0.9)	13.7 (0.9)	98.44 (0.07)
	NN	greedy	17.5 (3.3)	17.5 (3.3)	98.86 (0.15)
		UCB	18.4 (1.7)	18.4 (1.7)	98.89 (0.06)
		TS	19.0 (1.8)	19.0 (1.8)	98.95 (0.06)
	MPN	greedy	70.9 (4.1)	70.9 (4.1)	99.85 (0.03)
		UCB	93.9 (4.0)	93.9 (4.0)	99.97 (0.02)
		TS	82.2 (1.8)	82.2 (1.8)	99.91 (0.01)
0.2	RF	greedy	7.4 (2.0)	7.4 (2.0)	97.01 (0.73)
		UCB	12.2 (2.4)	12.2 (2.4)	98.12 (0.19)
		TS	8.1 (0.3)	8.1 (0.3)	97.35 (0.06)
	NN	greedy	8.5 (0.8)	8.5 (0.8)	97.90 (0.19)
		UCB	8.9 (1.9)	8.9 (1.9)	97.98 (0.20)
		TS	8.4 (1.0)	8.4 (1.0)	97.89 (0.11)
	MPN	greedy	38.3 (9.3)	38.3 (9.3)	99.46 (0.17)
		UCB	51.1 (8.9)	51.1 (8.9)	99.65 (0.11)
		TS	54.2 (4.3)	54.2 (4.3)	99.69 (0.05)
0.1	RF	greedy	3.5 (0.8)	3.5 (0.8)	96.04 (0.61)
		UCB	6.3 (0.6)	6.3 (0.6)	96.63 (0.24)
		TS	4.8 (0.6)	4.8 (0.6)	95.82 (0.21)
	NN	greedy	4.0 (0.6)	4.0 (0.6)	96.43 (0.39)
		UCB	5.0 (0.9)	5.0 (0.9)	96.64 (0.33)
		TS	4.6 (0.7)	4.6 (0.7)	96.63 (0.21)
	MPN	greedy	19.9 (1.5)	19.9 (1.5)	98.96 (0.09)
		UCB	26.3 (3.0)	26.3 (3.0)	99.17 (0.10)
		TS	25.3 (3.0)	25.3 (3.0)	99.11 (0.14)

Chemical Space Visualization

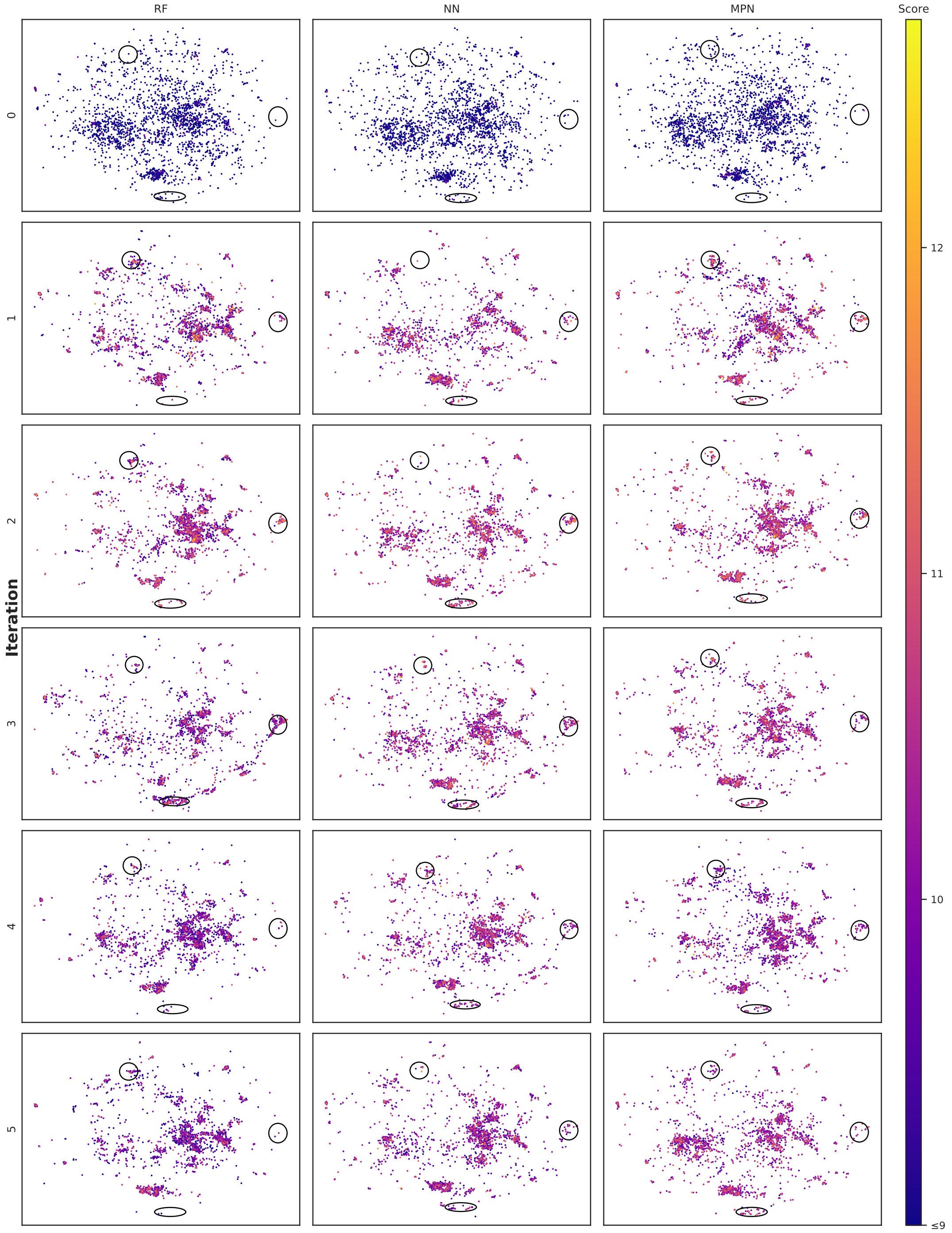

Figure S23: Visualization of the chemical space searched in the Enamine HTS library at the given iteration using a greedy acquisition metric, 0.1% batch size, and specified surrogate model architecture z-ordered by docking score. Points represent the 2D UMAP embedding of the given molecule's 2048-bit atom-pair fingerprint. The embedding was trained on a random 10% subset of the full library. Circled regions indicate clusters of high-scoring compounds in sparse regions of chemical space (Figure 8B). x- and y-axes are the first and second components of the 2D UMAP embedding and range from -7.5 to 17.5. Color scale corresponds to the negative docking score (higher is better).
